# Fast Ion-Chelate Dissociation Rate for *In Vivo* MRI of Labile Zinc with Frequency-Specific Encodability

**DOI:** 10.1021/jacs.1c05376

**Published:** 2021-07-23

**Authors:** Nishanth D. Tirukoti, Liat Avram, Talia Haris, Benjamin Lerner, Yael Diskin-Posner, Hyla Allouche-Arnon, Amnon Bar-Shir

**Affiliations:** † Department of Molecular Chemistry and Materials Science, Weizmann Institute of Science, Rehovot 7610001, Israel; ‡ Department of Chemical Research Support, Weizmann Institute of Science, Rehovot 7610001, Israel

## Abstract

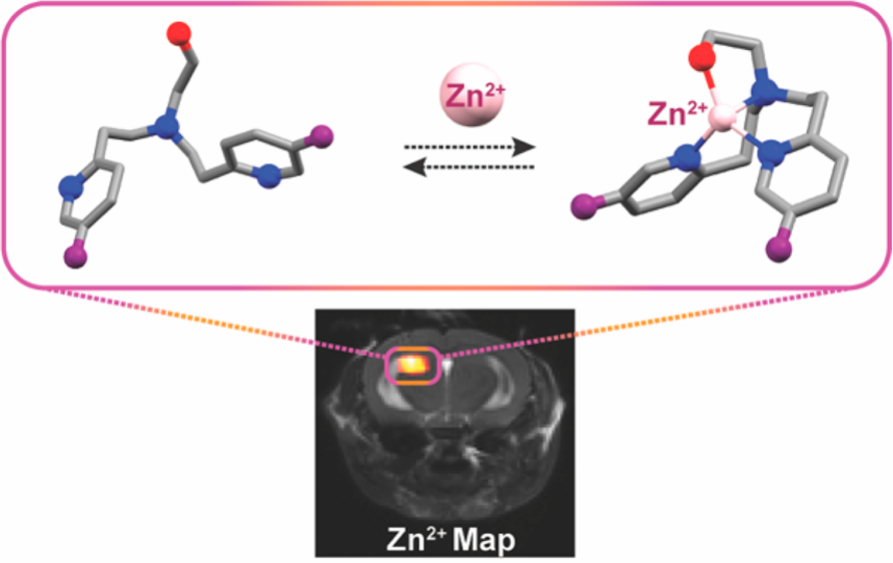

Fast ion-chelate dissociation rates and weak ion-chelate affinities are desired kinetic and thermodynamic features for imaging probes to allow reversible binding and to prevent deviation from basal ionic levels. Nevertheless, such properties often result in poor readouts upon ion binding, frequently result in low ion specificity, and do not allow the detection of a wide range of concentrations. Herein, we show the design, synthesis, characterization, and implementation of a Zn^2+^-probe developed for MRI that possesses reversible Zn^2+^-binding properties with a rapid dissociation rate (*k*_off_ = 845 ± 35 s^–1^) for the detection of a wide range of biologically relevant concentrations. Benefiting from the implementation of chemical exchange saturation transfer (CEST), which is here applied in the ^19^F-MRI framework in an approach termed ion CEST (iCEST), we demonstrate the ability to map labile Zn^2+^ with spectrally resolved specificity and with no interference from competitive cations. Relying on fast *k*_off_ rates for enhanced signal amplification, the use of iCEST allowed the designed fluorinated chelate to experience weak Zn^2+^-binding affinity (*K*_d_ at the mM range), but without compromising high cationic specificity, which is demonstrated here for mapping the distribution of labile Zn^2+^ in the hippocampal tissue of a live mouse. This strategy for accelerating ion-chelate *k*_off_ rates for the enhancement of MRI signal amplifications without affecting ion specificity could open new avenues for the design of additional probes for other metal ions beyond zinc.

## Introduction

Of the cations with a biological role, Zn^2+^ has garnered much interest due to (i) its involvement, as a tightly bound Zn^2+^, in the determination of the structure and activity of essential proteins^1^ and (ii) its role, as mobile Zn^2+^, in different secretion pathways of specific tissue.^2−5^ Labile Zn^2+^ was found in relatively large pools in the prostate’s epithelial cells,^6^ in the pancreatic β-cells,^7^ and in the hippocampal mossy fibers,^8^ and its distribution in these tissues was characterized using well-established Zn^2+^-specific fluorescent imaging probes.^9,10^ Extensively designed, these probes provide diverse affinity capabilities for Zn^2+^ to cover a wide range of cation concentrations, which varies between sub-nanomolar and millimolar in different tissues.^11−13^ Such variability in Zn^2+^ affinities was obtained through either replacing the commonly used dipicolyl amine amine (DPA)^14^ binding motif with other binding moieties (e.g., thioether,^15^ pyrrole,^16^ thiophen,^17^ quinoline,^18^ or pyrazine^19^) or by reducing the rigidity of the Zn^2+^-sensors.^20^ However, although this has increased our knowledge of Zn^2+^ biology, the need for multiple fluorescent probes to map wide and diverse concentrations of the cation, and the reduced specificity, poor readouts of probes with very low Zn^2+^ affinity (with a *K*_d_ at the mM level), and the limited depth penetration of fluorescent light, even of probes based on a long wavelength,^21^ call for further developments.

The advances in the design and implementation of MRI-responsive sensors have led to the development of sensors for spatially mapping the distribution of metal ions noninvasively from the deep tissues of live subjects,^22−29^ overcoming one of the major limitations of fluorescent-based probes. Specifically, for Zn^2+^, MRI-responsive agents have been in development for two decades using different strategies for MRI readout alternation,^30−40^ resulting in a few that were demonstrated *in vivo*. In addition to cell-penetrable formulations designed to image regions of rich pools of labile zinc in the brain of live animals,^22^ other formulations were used to map cell-secreted Zn^2+^ from both pancreatic^23^ and prostate^24^ tissues upon glucose stimulation, which showed, in real time, longitudinal modulation in the labile Zn^2+^ pools in live intact subjects. These agents were based on a DPA binding motif, which tightly binds Zn^2+^ with a *K*_d_ at the nM range, and were further developed as probes with a lower affinity for Zn^2+^ (at the μM range)^41−43^ to reduce background signals. Although this revolutionized the way secretory Zn^2+^ can be mapped upon external stimulation, further developments are still desired to grant MRI-responsive agents with much lower affinity capabilities, a wider range of Zn^2+^ concentration detectability, and spectrally resolved specificity.

A recent approach that combines ^19^F-MRI and chemical exchange saturation transfer (CEST)^44−47^ was developed to map metal ions and was thus termed ion CEST (iCEST). This approach shows several benefits over other MRI strategies for which responsive contrast agents are being developed. Among these are (i) the ability to provide spectrally resolved specificity based on the chemical shift of the ion-bound ligand; (ii) the advantages of fast ion-ligand dissociation rates toward enhanced signal amplifications; (iii) the ability to “turn on” the MRI contrast at will; and (iv) the use of a fluorinated probe that does not interfere with the anatomical MRI observations. Nevertheless, the iCEST probes used thus far to map Zn^2+^ secretion in a prostate cancer model *in vivo*(48) experience very strong cation affinity (*K*_d_ in the nM range), which is far from ideal for the preservation of basal cationic levels. Moreover, and very importantly, their slow ion-chelate dissociation rates (*k*_off_ = *k*_ex_ ≈ 20 s^–1^)^45^ result in a very low CEST signal enhancement, which further restricts, significantly, the dynamic range of concentrations of the ion that can be detected. Here, we show the design of a Zn^2+^-responsive MRI agent with improved readouts of low levels of the ion and NMR frequency-specificity compared to other competitive cations. Having rationalized a fluorinated chelate that weakly, but still with preserved high specificity, binds Zn^2+^, we were able to amplify signals from a wide range of biologically relevant concentrations of labile Zn^2+^ through reversible dynamic exchange, toward mapping pools of the cation in specific regions of the brain of a live animal, with no interference from competitive cations.

## Results and Discussion

**Figure 1:**
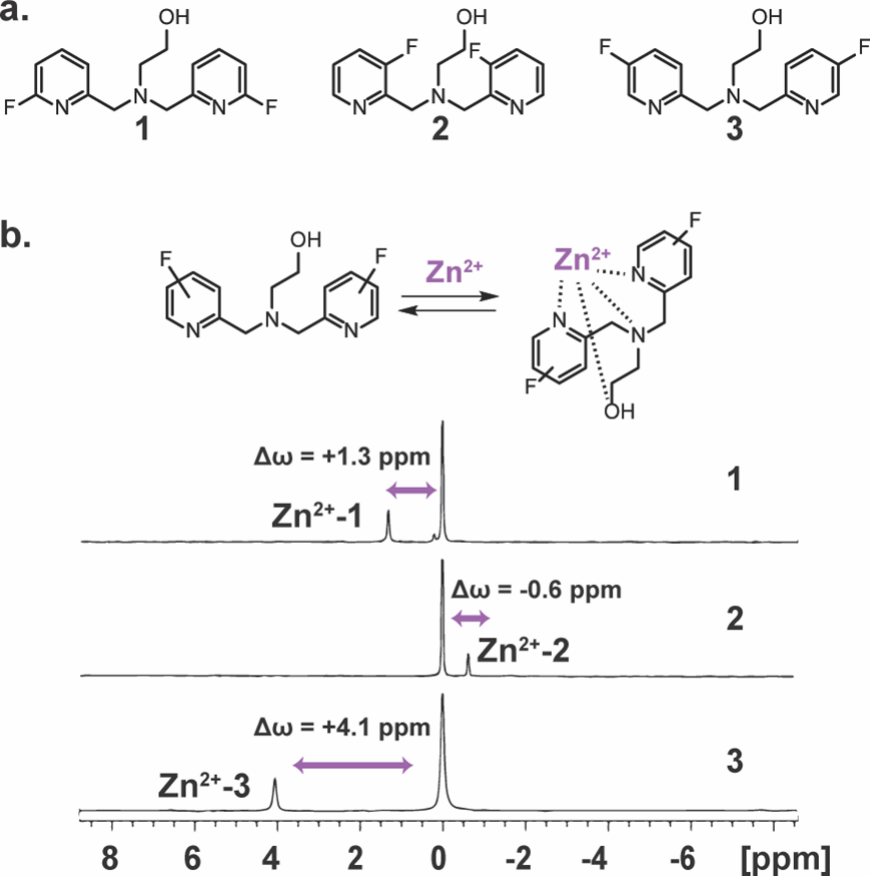
^19^F-NMR of fluorinated chelates designed for Zn^2**+**^ binding studies. (a) The chemical structure of the synthesized fluorinated chelates **1**, **2**, and **3** with the fluorine substituent at the 6, 3, and 5 positions of the pyridine ring, respectively. (b) Schematic illustration of the dynamic exchange process between the free and Zn^2+^-bound chelate and the obtained ^19^F-NMR spectrum of **1**, **2**, and **3** in the presence of Zn^2+^ at 25 °C (3 mM chelate and 0.6 mM ZnCl_2_ at 100 mM Hepes buffer, pH = 7.2, 9.4 T NMR). Shown are the chemical shift offsets (Δω) between the peak of the free chelate (set at 0.0 ppm) and the peak Zn^2+^-chelate complex.

The chemical shift offset (Δω) between two exchanging pools of spins is at the core of any designed CEST agent^49^ and thus, for ^19^F-CEST-based studies.^45,50−54^ This is mainly due to the fact that a larger Δω results in a reduced direct saturation effect, allowing the use of strong saturation powers for an enhanced CEST effect. Moreover, a larger Δω allows the use of CEST agents that are applicable under a faster exchange regime (fulfilling the condition Δω > *k*_ex_)^55^ and, therefore, are detectable at much lower concentrations.^56,57^ Thus, as a first step in our design, three putative fluorinated derivatives of DPA were synthesized based on the common use of a DPA backbone in both fluorescent-^7,8,14^ and MRI-^22,32^responsive probes developed for imaging labile Zn^2+^ under physiological conditions. To this end, employing a reductive amination using ethanolamine and different fluoropicolinaldehydes (see Supporting Information), the fluorinated chelates **1**, **2**, and **3** were synthesized with a fluorine substitution at positions 6, 3, and 5 of their pyridine rings, respectively (Figure 1a). Then, their ^19^F-NMR spectra in the presence of Zn^2+^ were examined to determine the Δω between free and Zn^2+^-bound chelate (Figure 1b). For all three examined chelates, an additional ^19^F-NMR peak that represented a Zn^2+^-bound chelate was depicted, with a characteristic Δω (relative to that of a free chelate set at 0.0 ppm) of +1.3, −0.6, or +4.1 ppm in the presence of **1**, **2**, and **3**, respectively.

**Figure 2:**
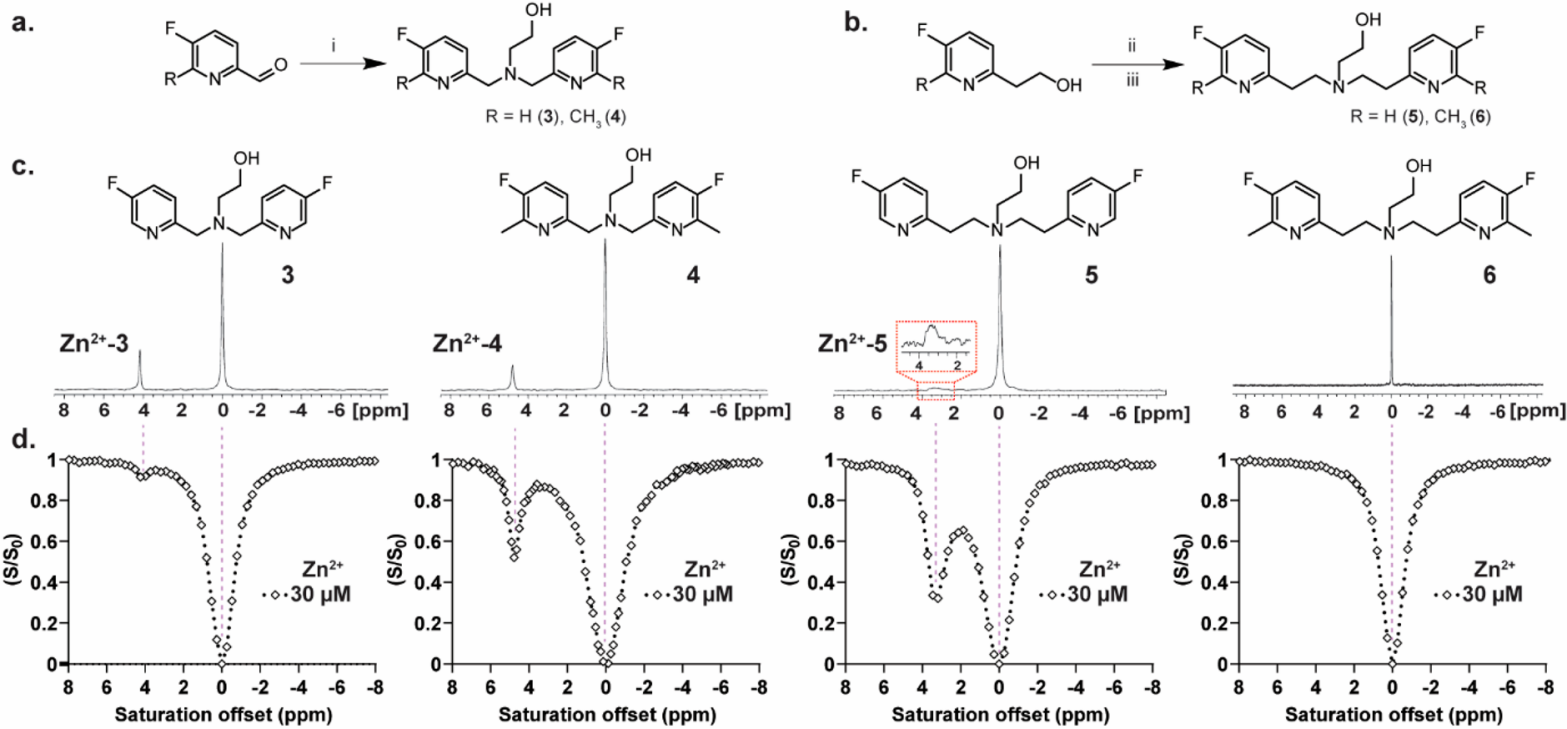
Zn^2+^-chelate exchange dynamics as a function of the chelate structure. (a) Synthetic route used for the synthesis of **3** and its methylated derivative **4**. (b) Synthetic route used for the synthesis of **5** and its methylated derivative **6**. (c) ^19^F-NMR spectra of 3 mM fluorinated chelates (**3**–**6**) in the presence of 0.6 mM Zn^2+^ at 37 °C and the obtained Δω between the peak of the free ligand (set at 0.0 ppm) and the Zn^2+^-bound ligand. (d) Representative ^19^F-iCEST spectra obtained for an aqueous solution of 3 mM of either of the chelates (from left to right: **3**, **4**, **5**, or **6**) in the presence of 30 μM Zn^2+^ at 37 °C. All NMR data were performed on aqueous solutions (100 mM Hepes buffer, pH = 7.2) at 37 °C with a 9.4 T NMR spectrometer. Reaction conditions: (i) 2-aminoethan-1-ol, NaBH(OAc)_3_; (ii) PPh_3_, CBr_4_; (iii) 2-aminoethan-1-ol, K_2_CO_3_.

Given that the largest Δω between free and Zn^2+^-bound chelate was identified for **3** (Δω = +4.1 ppm) with a fluorine substitution at position 5, we aimed to study the effect of the chelate structure on the obtained binding dynamic profile and the correspondent ^19^F-iCEST effect. To this end, another set of chelates was synthesized, namely, **4**, **5**, and **6** (Figure 2a and b), with the purpose of obtaining variable Zn^2+^-binding dynamics through the induction of a steric hindrance for Zn^2+^ binding (compare **3** to **4** and **5** to **6**) or by elongating the distance between the two pyridine rings of the chelate (compare **3** to **5** and **4** to **6**).^20^ The significant differences in the affinities of the four examined chelates to Zn^2+^ were manifested by the appearances of the ^19^F-NMR spectra of aqueous chelate:Zn^2+^ (3 mM:0.6 mM, which is a 5:1 ratio) solutions at 37 °C and a pH of 7.2 (Figure 2c).

While a narrow ^19^F-NMR peak was obtained for the **3**:Zn^2+^ complex, evidence of a very slow exchange rate in the NMR time scale, a broader and lower peak was obtained for the **4**:Zn^2+^ complex. The ^19^F-NMR peak of the **5**:Zn^2+^ was significantly broader and lower compared to that obtained for **4**:Zn^2+^ and **3**:Zn^2+^ complexes, indicative of the fast dissociation rate (*k*_off_, also termed the exchange rate, *k*_ex_, in CEST studies) between Zn^2+^-bound and free **5**. The absence of the ^19^F-NMR peak for the **6**:Zn^2+^ complex suggests that this system experiences very fast *k*_ex_ in the NMR time scale and, thus, is less likely to be considered as a ^19^F-iCEST sensor for Zn^2+^.

These differences in the ^19^F-NMR spectra of solutions of a Zn^2+^:chelate ratio of 1:5 were clearly reflected by the ^19^F-iCEST spectra obtained from solutions with reduced concentrations of the cation and a Zn^2+^:chelate ratio of 1:100 (Figure 2d). For example, in the presence 30 μM Zn^2+^ (3 mM chelate), only a 6% ^19^F-iCEST effect was obtained with probe **3**, which had increased to 45% for probe **4**. Such a dramatic signal amplification was enhanced even more when **5** was used as the putative probe. In the presence of 3 mM **5**, a very large ^19^F-iCEST effect of 62% was obtained for 30 μM Zn^2+^, implying a relatively fast *k*_ex_ between Zn^2+^-bound and free **5**. The absence of any ^19^F-iCEST effect for the solution of **6**:Zn^2+^ reflects a very fast *k*_ex_ between Zn^2+^-bound and free **6** (or lack of binding), beyond that required to obtain a robust CEST effect (*k*_ex_ ≤ Δω).

**Figure 3:**
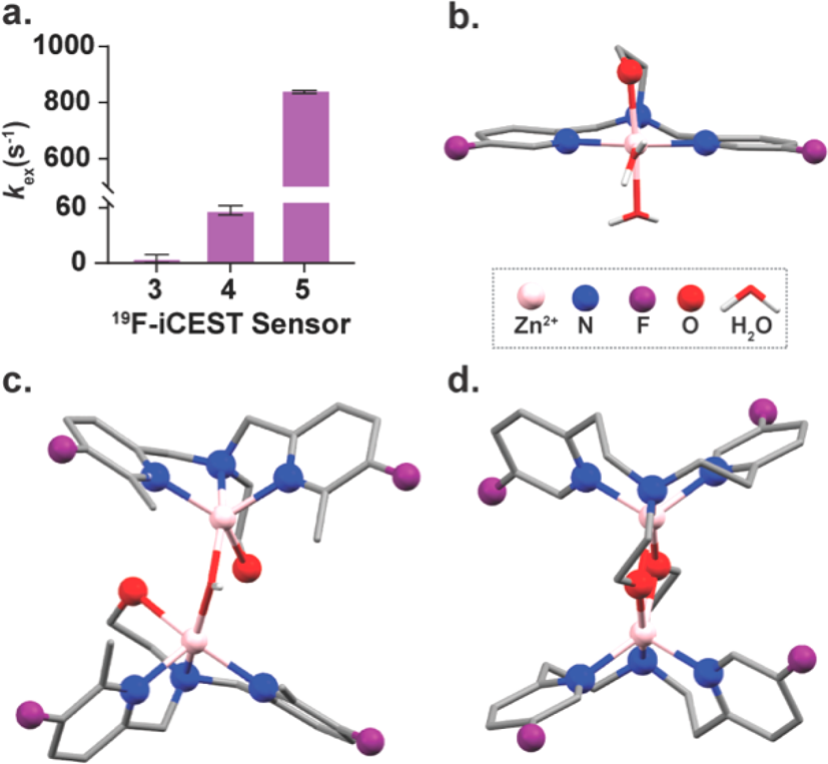
Zn^2+^-chelate exchange dynamics as a function of the chelate structure. (a) Evaluated exchange rates *k*_ex_ (s^–1^) between Zn^2+^-bound and free chelate as determined for **3**, **4**, and **5**. All NMR data were performed on aqueous solutions (100 mM Hepes buffer, pH = 7.2) at 37 °C with a 9.4 T NMR spectrometer. The X-ray crystal structures of the Zn^2+^-chelate complexes are shown for **3**-Zn^2+^ (b), **4**-Zn^2+^ (c), and **5**-Zn^2+^ (d).

Quantifying the *k*_ex_ values between Zn^2+^-bound and free chelate, for **3**–**5** (Figure 3a and Figure S1), further elaborated the relationship between the obtained ^19^F-iCEST effect, i.e., the signal amplification capabilities, and the kinetic properties of the complex. As qualitatively implied by the 1D-^19^F-NMR spectra (Figure 2c) and also reflected by the relatively low iCEST effect (Figure 2d), a very slow *k*_ex_ value (∼5 s^–1^) was obtained for **3**, as expected for a chelate that strongly binds Zn^2+^. The dissociation rate of Zn^2+^ from its bound state to the steric-hindered **4** was indeed faster (*k*_ex_ = 55 ± 5 s^–1^) and resulted in a more pronounced iCEST effect. The fastest *k*_ex_ value, i.e., the dissociation rate (*k*_off_) of chelate-bound Zn^2+^, was found for **5** (845 ± 35 s^–1^), which explains the very weak and broad ^19^F-NMR peak of the Zn^2+^-**5** complex (Figure 2c), which also translated to the largest ^19^F-iCEST effect (Figure 2d). These results show that, by introducing chemical modification to fluorinated chelates, we can modulate the ion-chelate binding kinetics characteristics (obtaining relatively fast *k*_off_) while preserving the spectral specificity of the bound cation (Δω of +3.2 ppm for the Zn^2+^-**5** complex).

To further elaborate on the linkage between the structure of the obtained Zn^2+^ complex and its obtained kinetic properties, we aimed to crystallize the complexes of Zn-**3**, Zn-**4**, and Zn-**5**, which clearly reflected different chelating properties of the three fluorinated probes (Figure 3b–d and Supporting Information). In the obtained crystal of Zn-**3**, Zn^2+^ adopts an octahedral arrangement with coordination to three nitrogen atoms of the DPA motif, to the hydroxyl oxygen of the side arm, and to two water molecules (Figure 3b). The crystal structures of Zn-**4** and Zn-**5** revealed two different dimers of five-coordinate Zn^2+^ complexes. In both complexes, Zn^2+^ is coordinated to the three nitrogen atoms of the DPA motif and to the hydroxyl oxygen side arm. In the Zn-**4** dimer both Zn^2+^ ions share coordination to the same water molecule (Figure 3c).

In the Zn-**5** dimer both Zn^2+^ ions share a coordination to the hydroxyl side arm of itself and its adjacent neighbor (Figure 3d), which may further explain, in addition to the flexibility of the chelate achieved by its longer distance between the pyridine moieties, the loosened binding of Zn^2+^ to **5** and the obtained faster *k*_off_. The low binding affinity of **5** to Zn^2+^ (*K*_d_ = (5.5 ± 0.6) × 10^–3^ M, Table S1 and Figure S2), which is a result of chelating properties observed from the crystal structure of the Zn-**5** complex (Figure 3d), is preferable to maintain the steady-state concentration of the cation in the studied region and to prevent its dissociation from proteins, where it plays a critical role in both structure and function.

**Figure 4:**
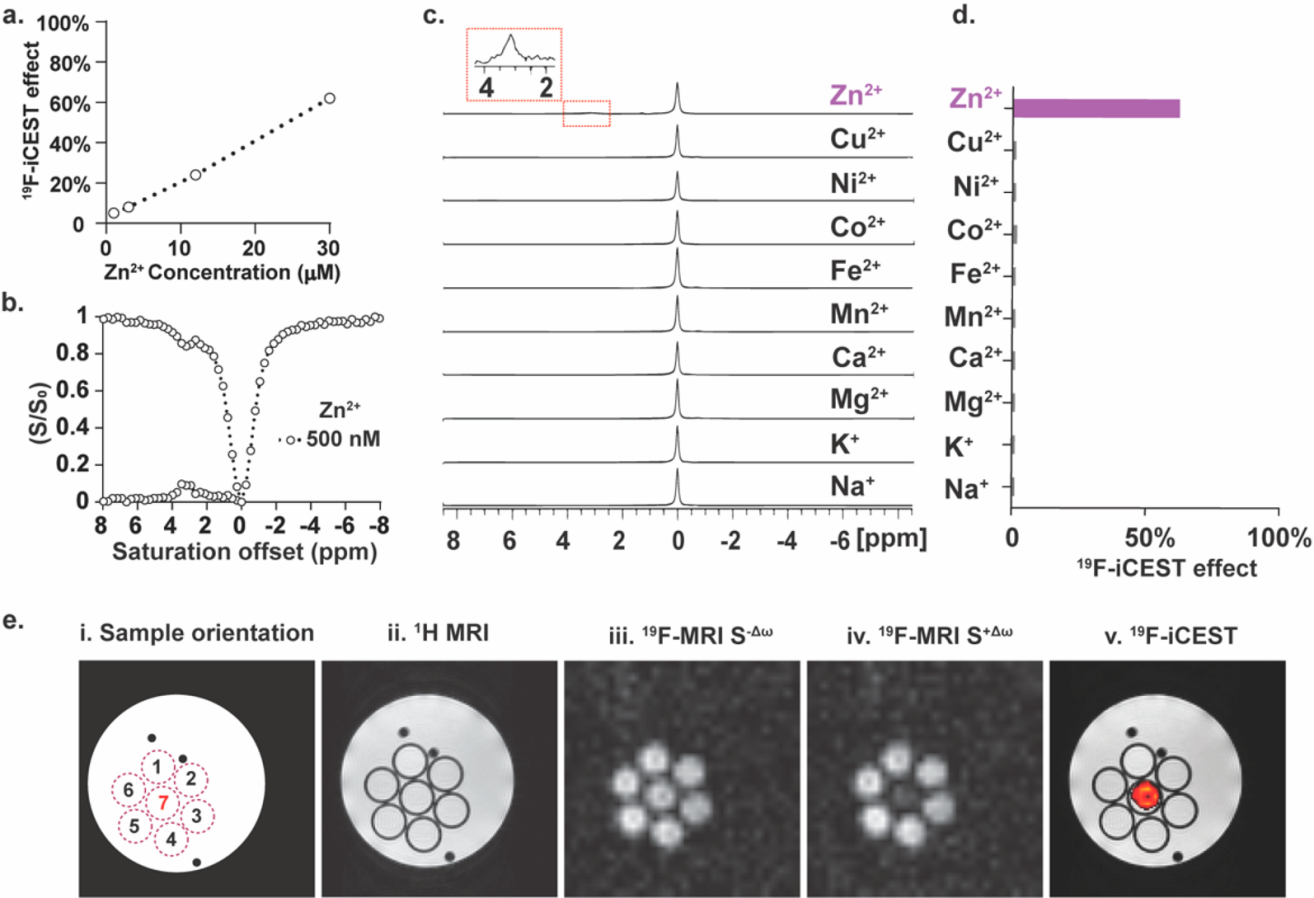
^19^F-iCEST Zn^2**+**^ sensitivity and selectivity using **5**. (a) ^19^F-iCEST effect for 3 mM **5** as a function of Zn^2+^ concentration. (b) ^19^F-iCEST profile of 1 mM **5** and 500 nM Zn^2+^. (c) ^19^F-NMR spectra of 3 mM **5** in the presence of 0.6 mM Na^+^, K^+^, Mg^2+^, Ca^2+^, Mn^2+^, Fe^2+^, Co^2+^, Ni^2+^, Cu^2+^, and Zn^2+^ at 25 °C. (d) ^19^F-iCEST effect (Δω = 3.2 ppm) of 3 mM **5** and 30 μM (in 100 mM Hepes buffer, pH = 7.2) of s-block (Na^+^, K^+^, Mg^2+^, Ca^2+^) and d-block (Mn^2+^, Fe^2+^, Co^2+^, Ni^2+^, Cu^2+^, Zn^2+^) metal ions obtained at 37 °C on a 9.4 T NMR spectrometer. (e) ^19^F-iCEST MRI: (i) schematic representation of the studied phantom composed of seven tubes containing 7 mM **5** and 100 μM cation, i.e., Ca^2+^ (#1), Cu^2+^ (#2), Mg^2+^ (#3), Na^+^ (#4), K^+^ (#5), and Zn^2+^ (#7). Tube #6 contained only **5**; (ii) ^1^H-MRI; (iii) ^19^F-MRI obtained with a presaturation pulse applied at Δω = −3.2 ppm; (iv) ^19^F-MRI obtained with a presaturation pulse applied at Δω = +3.2 ppm; (v) ^19^F-iCEST map obtained by the subtraction of the image in (iv) from that in (iii) overlaid on ^1^H-MRI.

Since **5** was identified as the fluorinated chelate with the preferred characteristics (Figures 2 and 3), it was used in a set of ^19^F-iCEST experiments with a range of Zn^2+^ concentrations (1–30 μM, Figure 4a). Indeed, as a result of its fast *k*_off_ (*k*_ex_ = 845 ± 35 s^–1^), the use of **5** provided the ability to detect a relatively wide range of Zn^2+^ concentrations with a conventional ^19^F-MR setup based on the amplification principle of CEST, which depends, among other parameters, on *k*_ex_.

For example, a 5% ^19^F-iCEST effect was obtained for 1 μM Zn^2+^ in the presence of 3 mM **5** (3000:1 ratio of chelate:ion), which corresponds to a ×150 signal amplification. The fact that one can control the concentration of the ^19^F-iCEST probe in the solution allows detection of even lower concentrations of Zn^2+^. In this regard, by reducing the concentration of **5** to 1 mM, a 10% effect in the presence of 500 nM Zn^2+^ could be detected at the expected frequency (Figure 4b). This is one major advantage of iCEST over water proton-based CEST agents, in analogy to the hyperCEST-based approach,^58,59^ where the bulk pool of free imaging agent (the ^19^F-MR signal of **5** in this study) is controllable and very small in comparison to the bulk water signal in tissue. Thus, although the main limitation of iCEST is its sensitivity that is based on ^19^F-MR and therefore relies on the deliverable amount of **5** into the studied region, this approach allows the detection of very low concentrations of cations with no background signal from the surrounding tissue.

Given that **5** was identified as the preferable ^19^F-iCEST agent for labile Zn^2+^, its specificity to detect this cation was examined. To this end, the ^19^F-iCEST effect of **5** in the presence of competitive ions, either those that are abundant in biological systems (e.g., Na^+^, K^+^, Mg^2+^, or Ca^2+^) or those that might have shared similar affinities to a Zn^2+^ chelate (Fe^2+^, Mn^2+^, Cu^2+^), was studied. Importantly, even though **5** binds Zn^2+^ with reduced affinity, neither additional chelate-ion complex peaks in ^19^F-NMR studies (Figure 4c) nor a ^19^F-iCEST effect was obtained for this chelate in the presence of the other studied cations (Figure 4d and Figure S3). Such an ability to detect Zn^2+^ with ultimate specificity, which is manifested by a characteristic ^19^F-iCEST spectrum with a spectrally resolved ^19^F-CEST peak (at Δω = +3.2 ppm), is an advantage of the proposed platform over commonly used and very sensitive relaxation-based MRI agents that do not possess this unique feature. To further demonstrate this, a phantom composed of test tubes that contained different cations in the presence of **5** was prepared and studied using a 9.4 T MRI scanner (Figure 4e). As expected, the presence of **5** did not affect the overall ^1^H MRI contrast of the studied solution, implying on no effect on anatomical MR observation in *in vivo* studies. Similarly, when the saturation pulse was applied “off-resonance”, i.e., Δω = −3.2 ppm from the ^19^F-NMR frequency of **5**, no difference could be detected when comparing the ^19^F-MRI signals of the examined tubes. Nevertheless, when the saturation pulse was applied at Δω = +3.2 ppm, the frequency offset of the **5**-Zn^2+^ complex (Figure 2c and d and Figure S4), a clear reduction of the ^19^F-MRI signal of the tube containing the Zn^2+^ ion (center tube) could be depicted. This manifestation of frequency-specific detectability of the cation of interest is presented as a ^19^F-iCEST contrast map that could be further overlaid on ^1^H-MRI for the spatial display of Zn^2+^ in the examined tube.

**Figure 5:**
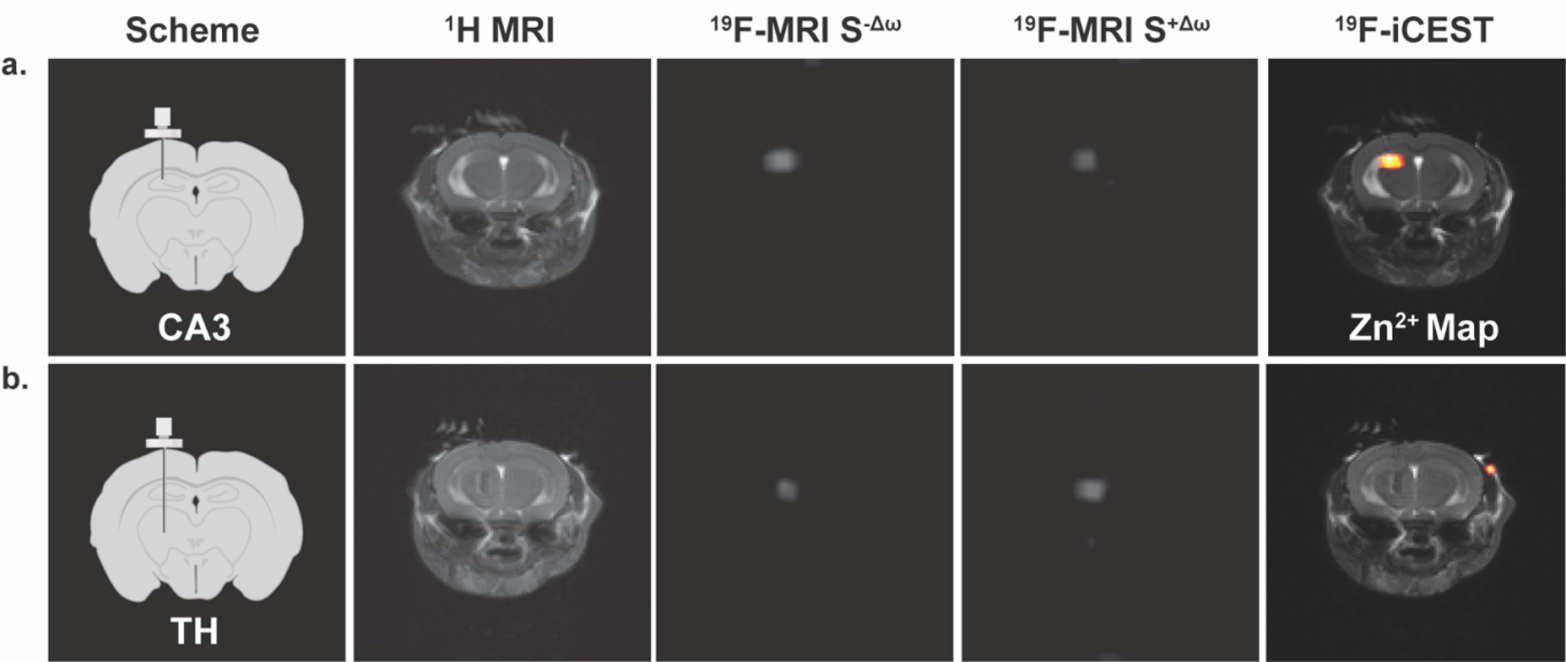
*In vivo*^19^F-iCEST maps of labile Zn^2+^ pools in the mouse brain. Shown results for two regions of the brain: (a) CA3 in the hippocampus (zinc-rich ROI) or (b) the thalamus (TH, zinc-poor ROI). From left-to-right are the schematic illustration of the setup used to deliver **5** to either CA3 or TH, the^1^H-MRI, the^19^F-MRI S^–Δω^ (presaturation pulse applied at Δω = −3.2 ppm, i.e., “off-resonance”), the ^19^F-MRI S^+Δω^ (presaturation pulse applied at Δω = +3.2 ppm, i.e., “on-resonance”), and the ^19^F-iCEST contrast (Zn^2+^ map) obtained from subtracting ^19^F-MRI S^+Δω^ from ^19^F-MRI S^–Δω^ overlaid on the ^1^H-MRI. MRI scans were performed at 15.2 T. Infusion rate was set to 0.25 μL/min (of 10 mM **5** in PBS), and iCEST data acquisition started 90 min from the onset of the infusion of **5**.

Then, we examined the potential of **5** to be used for *in vivo* mapping of labile zinc pools. For that purpose, two different regions of the brain, which are known for their different endogenous labile Zn^2+^ levels, were targeted. The CA3 region of the hippocampus was chosen as a region-of-interest (ROI) that is rich with labile Zn^2+^ and the thalamus (TH) as an ROI with very low levels of labile Zn^2+^.^60^ After evaluating its biocompatibility, even at the high concentrations needed for ^19^F-MRI (Figure S5), and showing that the lipophilicity of **5** allows its intracellular delivery (Figure S6), it was delivered intracranially to either CA3 (Figure 5a) or TH (Figure 5b) through a continuous infusion in order to compensate for its fast washout from the brain (Figure S7). Ninety minutes from starting the infusion (0.25 μL/min), when the concentration of **5** was estimated to be 1.2 mM in the studied region (Figure S8), ^19^F-MRI data sets were acquired with a presaturation pulse applied at either “off-resonance” (Δω = −3.2 ppm) or “on-resonance” (Δω = +3.2 ppm). The ^19^F-iCEST maps were then derived by subtraction of the ^19^F-MRI obtained “on-resonance” from that obtained “off-resonance”. As expected from a labile-zinc-rich ROI (CA3, Figure 5a), a significant ^19^F-iCEST effect was obtained, which was represented as a Zn^2+^ map overlaid on a ^1^H-MRI anatomical view (Figure 5a, right). In contrast, when **5** was delivered to a region that was not expected to have high levels of labile Zn^2+^ (TH, Figure 5b), no pronounced difference between the two ^19^F-MRI data sets could be depicted and the obtained ^19^F-iCEST showed no signal (Figure 5b, right).

**Figure 6:**
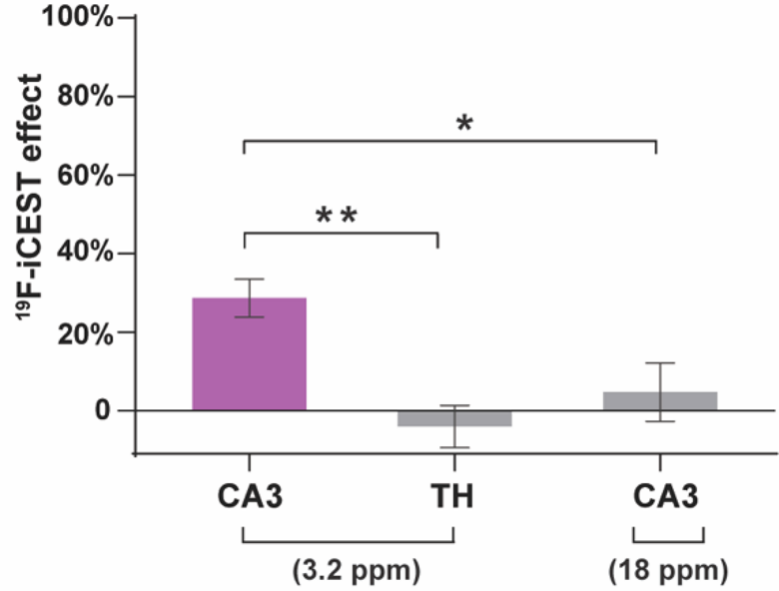
*In vivo*^19^F-iCEST quantification plot: The average percentile of ^19^F-iCEST contrast (S^Δω+^/S^Δω–^) as quantified in CA3 at Δω = 3.2 ppm (*N* = 7 mice) or Δω = 18 ppm (*N* = 7 mice), or in the TH (*N* = 7) at Δω = 3.2 ppm. Error bar denotes SEM, *p-value < 0.05, **p-value < 0.001, unpaired Student’s *t* test.

Quantifying the obtained results from a group of mice showed a significant difference between the ^19^F-iCEST effect for the two regions (Figure 6, CA3 vs TH, *N* = 7/group, *p*-value < 0.001), with an average 29 ± 5% signal change in the labile-Zn^2+^-rich ROI, CA3. Importantly, when the presaturation pulse was applied at Δω = ±18 ppm, no observable ^19^F-iCEST effect was depicted, even in CA3 (*N* = 7, Figure 6 and Figure S9), confirming that a significant effect is obtained only from a zinc-rich region (CA3) and only when the saturation pulse is applied at a specific frequency (Δω = +3.2 ppm).

## Conclusions

In conclusion, we showed here the design of a fluorinated chelate (**5**), which features a fast Zn^2+^-chelate dissociation rate and can be used for *in vivo* MRI mapping of labile Zn^2+^ with improved sensitivity and supreme specificity. Obtaining a fast *k*_ex_ of 845 ± 35 s^–1^, at which Zn^2+^-bound and unbound states of **5** exchanged, provided the capability to detect a wide range of the cation concentrations that can be mapped using a single molecular probe. This is in contrast to fluorescent probes, where multiple probes are needed to cover the expected concentrations, with high-affinity probes useful for mapping low pools of labile zinc, while low-affinity probes are a better fit for imaging high concentrations of the cation.^20^ Moreover, and in contrast to other imaging strategies where low binding affinities can compromise both the specificity over other competitive cations and the signal readout changes (i.e., contrast-to-noise ratio) upon ion-binding, we demonstrated that the weak binding of Zn^2+^ to **5** did not affect its Zn^2+^ specificity or detectability. Having demonstrated the ability to map labile Zn^2+^ pools in a deep tissue of live animals, the proposed ^19^F-iCEST approach should be further applied to study dynamic changes in the cation concentration as a result of external stimulation or as a result of pathological events.^23,24,42,48^ Although demonstrated here for Zn^2+^ imaging, the principles outlined in this work should be further extended to rationalize the design of new ^19^F-iCEST probes to detect other metal ions with biological relevance and significance,^61^ especially those that may be found either at very low concentrations or in a wide range of concentrations where multiple probes with different binding affinities are still needed.

## Supporting Information Available

Chemical synthesis procedures, NMR and MRI studies, exchange rate (*k*_ex_) evaluations, dissociation constant (*K*_d_) measurements, crystallization protocol and crystal structure data, cell viability assay and *in vivo* experiments (PDF)

## Accession Codes

CCDC 2084667–2084671 contain the supplementary crystallographic data for this paper. These data can be obtained free of charge via www.ccdc.cam.ac.uk/data_request/cif, or by emailing data_request@ccdc.cam.ac.uk, or by contacting The Cambridge Crystallographic Data Centre, 12 Union Road, Cambridge CB2 1EZ, UK; fax: +44 1223 336033.
