# Supplementary material for: Fast Ion-Chelate Dissociation Rate for In Vivo MRI of Labile Zinc with Frequency-Specific Encodability
Source: J Am Chem Soc. 2021 Jul 23;143(30):11751–8. doi: 10.1021/jacs.1c05376 (PMC8397314; doi:10.1021/jacs.1c05376)
Supplement: Supplementary file 1 — ja1c05376_si_001.pdf [file ja1c05376_si_001.pdf]

Supporting information:

Fast Ion-Chelate Dissociation Rate for *In Vivo* MRI of Labile Zinc with Frequency-Specific Encodability

Nishanth D. Tirukoti [1], Liat Avram[2], Talia Haris[2], Benjamin Lerner[1], Yael Diskin-Posner[2], Hyla Allouche-Arnon[1], Amnon Bar-Shir[1]*

1Department of Molecular Chemistry and Materials Science, Weizmann Institute of Science, Rehovot 7610001, Israel.

2Department of Chemical Research Support, Weizmann Institute of Science, Rehovot 7610001, Israel.

*Corresponding Author: amnon.barshir@weizmann.ac.il

Table of contents

A. Materials and methods……………………………………………………………………..S2

B. Chemical synthesis procedure ………………………………………………………………S3

C. ^{19}F-iCEST experiments ……………………………………………………………………...S8

D. Bloch-McConnell data fitting ………………………………………………………………S8

E. Binding constant (K_d) measurements by ^{19}F NMR………………………………………S8

F. Crystallization procedure ……………………………………………………………….....S9

G. Cell viability ……………………………………………………………………………...S9

H. MRI experiments ……………………………………………………………………….S10

I. Supporting information Figures …………………………………………………………..S12

J. NMR (^{1}H, ^{13}C, ^{19}F) & HRMS Spectra ………………………………………………....S17

K. Crystallographic data………………………………………………………………………..S29

L. Supplementary references…………………………………………………………......S33

A. Materials and Methods

All the reagents and solvents were obtained from commercial suppliers. Fluoropicolinaldehydes, such as 3- fluoropicolinaldehyde, 5-fluoropicolinaldehyde, and 6- fluoropicolinaldehyde, were purchased from Oxchem Corporation Chemicals (Wood Dale, IL). 5- fluoro-6-methylpicolinaldehyde, 2-(5-fluoropyridin-2-yl)ethan-1-ol, and 2-(5-fluoro-6-methylpyridin-2-yl)ethan-1-ol were purchased from Pharma Block Sciences, Inc. (Nanjing, China). Sodium triacetoxyborohydride and sodium sulphate were purchased from Alfa Aesar. Dipicolylamine (DPA) was purchased from Sigma Aldrich. Deuterated solvents, such as $CDCl_3$ and D_2O, were purchased from Cambridge Isotope Laboratories, Inc. (Andover, MA). The ^{1}H NMR, ^{13}C NMR, ^{19}F NMR spectra, ^{19}F-NMR Zn^{2+} binding studies, and ^{19}F-iCEST experiments were performed on a Bruker AVANCE III 9.4 T NMR spectrometer. The following abbreviations are used to describe peaks: s-singlet; d- doublet; t-triplet; m- multiplet. High-resolution mass spectrometry (HR-MS) was recorded on an AB SCIEX 5800 MALDI TOF instrument at the Weizmann institute of Science mass spectrometry facility. Phantom magnetic resonance imaging (MRI) experiments were performed on a 9.4 T wide-bore MR scanner (Bruker AVANCE III system). A 25 mm, double-resonant ($^{1}H/^{19}F$) radiofrequency (RF) coil was used to acquire ^{1}H, ^{19}F, and ^{19}F-iCEST MR images. For animal surgeries, a customized stereotaxic surgery setup was used, with 2 mm and 3.5 mm MRI-compatible cannulas that were purchased from p1 Technologies (Roanoke, Virginia, USA). Animal MRI experiments were performed on a Bruker BioSpec 15.2 T AVANCE III HD imaging spectrometer. A dual $^{1}H/^{19}F$, 23 mm RF coil was used to acquire the ^{1}H, ^{19}F-MR, and ^{19}F-iCEST images. For the details of X-ray data collection and refinement, see section K.

B. Chemical Synthesis procedure

Compound 1:

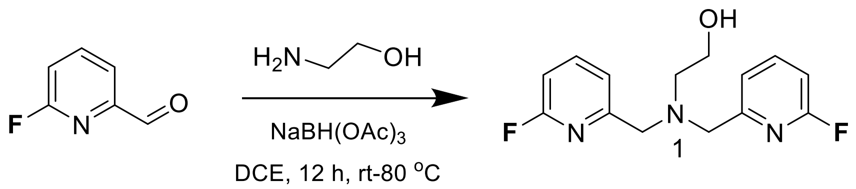

6-fluoropicolinaldhyde (166.40 mg, 1.33 mmol) was added to a solution of 2-aminoethan-1-ol (32.5 mg, 532.05 µmol) in 1,2-dichloroethane (DCE) (8 mL) and stirred at room temperature for 30 min followed by the addition of sodium triacetoxyborohydride (338 mg, 1.59 mmol). The reaction mixture was then stirred at room temperature for an additional 1 h before being heated to 80 °C for 12 h. Completion of the reaction was confirmed by thin layer chromatography (TLC) and the solvent was evaporated under reduced pressure. The crude product was dissolved in $CHCl_3$, washed with a saturated solution of $NaHCO_3$ and extracted with $CHCl_3$ (2×20 mL), then the combined organic phase was dried over Na_2SO_4. Following filtration, the organic phase was concentrated and purified using silica gel column chromatography ($CHCl_3$/MeOH, 95:5) to obtain compound **1** as a pale yellow oil (122 mg, 83%). ^{1}H NMR (400.35 MHz, $CDCl_3$): δ 7.72 (m, 2H), 7.28 (d, $J_{H\text{-}F}$ =7.5 Hz, 2H), 6.78 (d, $J_{H\text{-}F}$=8.4 Hz, 2H), 3.86 (s, 4H), 3.66 (t, J=5.2 Hz, 2H), 2.84 (t, J=5.2 Hz, 2H). ^{13}C{^{1}H} (100.67 MHz, $CDCl_3$) δ 163.0 (d, $J_{C\text{-}F}$ = 240 Hz), 158.1 (d, $J_{C\text{-}F}$ = 12.4 Hz), 141.5 (d, $J_{C\text{-}F}$ = 7.7 Hz), 120.2 (d, $J_{C\text{-}F}$ = 4.1 Hz), 107.7 (d, $J_{C\text{-}F}$ = 36.5 Hz), 59.2, 59.1, 56.5. ^{19}F (376.7 MHz, $CDCl_3$) δ -68.25 (m). HRMS (ESI) calculated for $C_{14}H_{15}F_2N_3NaO$ [M+Na] m/z 302.1081, found m/z 302.1087.

Compound 2:

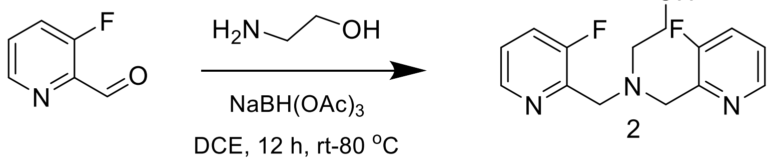

3-fluoropicolinaldhyde (101.5 mg, 0.881 mmol) was added to a solution of 2-aminoethan-1-ol (20 mg, 372 µmol) in 1,2-dichloroethane (5 mL) and stirred at room temperature for 30 min followed by the addition of sodium triacetoxyborohydride (316 mg, 1.1 mmol). The reaction mixture was then stirred at room temperature for an additional 1 h before being heated to 80 ^{o}C for 12 h. Completion of the reaction was confirmed by TLC and the solvent was evaporated. The crude product was dissolved in $CHCl_3$, washed with a saturated solution of $NaHCO_3$ and extracted with $CHCl_3$ (2×20 mL), and the combined organic phase was dried over Na_2SO_4. Following filtration, the organic phase was concentrated and purified using a silica gel column ($CHCl_3$/MeOH, 95:5) to obtain compound **2** as a pale-yellow oil (81 mg, 90%). ^{1}H NMR (400.35 MHz, $CDCl_3$): δ 8.42 (m, 2H), 7.39 (m, 2H), 7.27 (m, 2H), 4.14 (m, 4H), 3.7 (t, *J*=5 Hz, 2H), 2.97 (t, *J*=5 Hz, 2H). ^{13}C{^{1}H} (100.67 MHz, $CDCl_3$) δ 158.2 (d, J_{C-F} = 258.8 Hz), 147.1 (d, J_{C-F} = 14.1 Hz), 144.6 (d, J_{C-F} = 5.7 Hz), 123.6 (d, J_{C-F} = 3.7 Hz), 122.9 (d, J_{C-F} = 20 Hz), 59.3, 56.1, 53.6. ^{19}F (376.7 MHz, $CDCl_3$) δ -125.74 (d, J_{F-H} = 8.7 Hz). HRMS (ESI) calculated for $C_{14}H_{16}F_2N_3O$ [M+H] *m/z* 280.1261, found *m/z* 280.1265.

Compound 3 :

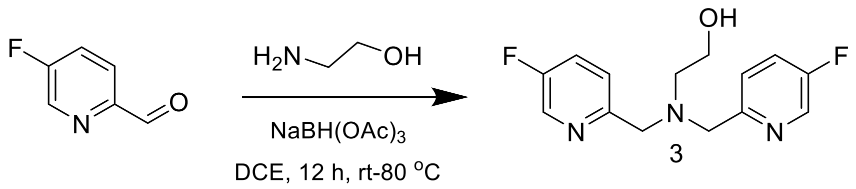

5-fluoropicolinaldhyde (180 mg, 1.43 mmol) was added to a solution of 2-aminoethan-1-ol (35 mg, 372 µmol) in 1,2-dichloroethane (10 mL) and stirred at room temperature for 30 min followed by the addition of sodium triacetoxyborohydride (362 mg, 1.7 mmol). The reaction mixture was then stirred at room temperature for an additional 1 h before being heated up to 80 ^{o}C for 12 h. Completion of the reaction was confirmed by TLC and the solvent was

evaporated. The crude product was dissolved in $CHCl_3$, washed with a saturated solution of $NaHCO_3$ and extracted with $CHCl_3$ (2×20 mL), and the combined organic phase was dried over Na_2SO_4. Following filtration, the organic phase was concentrated and purified using a silica gel column ($CHCl_3$/MeOH, 95:5) to obtain compound **3** as a pale white yellowish solid (144 mg, 90%). ^{1}H NMR (400.35 MHz, $CDCl_3$): δ 8.46 (s, 2H), 7.40 (m, 4H), 3.90 (s, 4H), 3.67 (t, *J*=5.1 Hz, 2H), 2.85 (t, *J*=5.1 Hz, 2H). $^{13}C\{^1H\}$ (100.7 MHz, $CDCl_3$) δ 158.6 (d, J_{C-F} = 255 Hz), 155.1 (d, J_{C-F} = 3.4 Hz), 137.1 (d, J_{C-F} = 23.6 Hz), 123.9 (d, J_{C-F} = 4.18 Hz), 123.3 (d, J_{C-F} = 18.3 Hz), 59.5, 59.2, 56.5 ^{19}F (376.7 MHz, $CDCl_3$) δ -130.6 (t, J_{F-H} = 6 Hz). HRMS (ESI) calculated for $C_{14}H_{16}F_2N_3O$ [M+H] *m/z* 280.1261, found *m/z* 280.1268.

Compound 4:

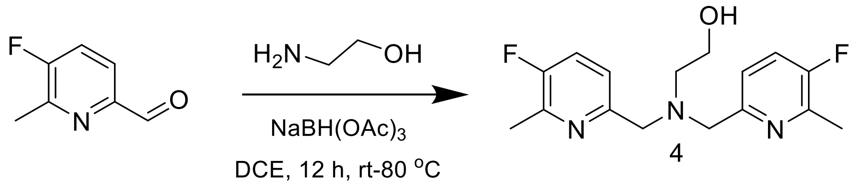

5-fluoro-6-methylpicolinaldhyde (171 mg, 1.23 mmol) was a added to a solution of 2-aminoethan-1-ol (30 mg, 491 μmol) in 1,2-dichloroethane (10 mL) and stirred at room temperature for 30 min followed by the addition of sodium triacetoxyborohydride (310 mg, 1.4 mmol). The reaction mixture was then stirred at room temperature for an additional 1 h before being heated to 80 °C for 12 h. Completion of the reaction was confirmed by TLC and the solvent was evaporated. The crude product was dissolved in $CHCl_3$, washed with a saturated solution of $NaHCO_3$ and extracted with $CHCl_3$ (2×20 mL), and the combined organic phase was dried over Na_2SO_4. Following filtration, the organic phase was concentrated and purified using silica gel column chromatography ($CHCl_3$/MeOH, 97:3) to obtain compound **4** as a pale yellow solid (138 mg, 92%). ^{1}H NMR (400.35 MHz, $CDCl_3$): δ 7.32 (m, 2H), 7.24 (m, 2H), 3.93 (s, 4H), 3.74 (t, *J*=4.9 Hz, 2H), 2.92 (t, *J*=4.9 Hz, 2H), 2.59 (s, 6H). $^{13}C\{^1H\}$ (100.67 MHz, $CDCl_3$) δ 156.9 (d, J_{C-F} = 253.3 Hz), 154.0 (d, J_{C-F} = 5.05 Hz), 145.9 (d, J_{C-F} = 17 Hz), 122.7 (d, J_{C-F} = 19.6 Hz), 121.5 (d, J_{C-}

$_F$ = 3.75 Hz), 59.6, 59.3, 56.6, 17.8. ^{19}F (376.7 MHz, $CDCl_3$) δ -128.60 (m). HRMS (ESI) calculated for $C_{16}H_{19}F_2N_3NaO$ [M+Na] *m/z* 330.1394, found *m/z* 330.1400.

Compound 5a, 5:

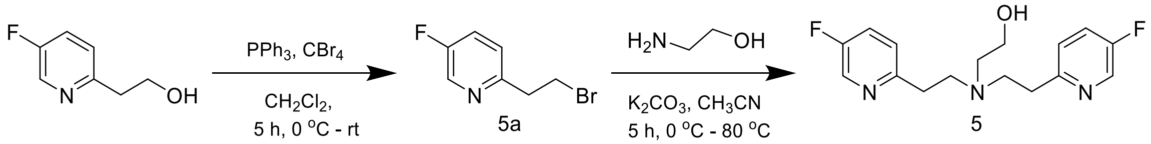

5a : To a solution of 2-(5-fluoropyridin-2-yl)ethan-1-ol (250 mg, 1.77 mmol) in CH_2Cl_2 (5 mL), PPh_3 (1.39 g, 5.31 mmol) was added followed by CBr_4 (1.76 g, 5.31 mmol) at 0 °C. The reaction mixture was stirred at room temperature for 2 h. Completion of the reaction was confirmed by TLC, solvent was removed under reduced pressure and the resulting residue was purified by silica gel column chromatography (EtOAc/Petroleum ether, 5:95) to obtain compound **5a** as yellow oil (260 mg, 72%). LC/MS (ES, *m/z*) [M+H]: 203.

5 : Compound **5a** (260 mg ,1.27 mmol) was added to a solution of 2-aminoetha-1-ol (31 mg, 507.50 μmol) in CH_3CN (3 mL) and stirred for 5 min under N_2 at room temperature. This was followed by addition of potassium carbonate (210 mg, 1.52 μmol), then the reaction mixture was heated to 80 °C for 5 h. Completion of the reaction was confirmed by TLC and the solvent was evaporated under reduced pressure. The crude product was dissolved in $CHCl_3$, washed with brine solution and extracted with $CHCl_3$ (2×20 mL), and the combined organic phase was dried over Na_2SO_4. Following filtration, the organic phase was concentrated and purified using a silica gel column ($CHCl_3$/MeOH 95:5) to obtain compound **5** as yellow oil (120 mg, 77%). ^{1}H NMR (400.35 MHz, $CDCl_3$): δ 8.41 (d, $J_{H\text{-}F}$ = 2.79 Hz, 2H), 7.3 (m, 2H), 7.0 (m, 2H), 3.62 (t, J = 5 Hz, 2H), 2.99 (t, J = 6.2 Hz, 4H), 2.91 (t, J = 6.2 Hz, 4H), 2.79 (t, J = 5 Hz, 2H). ^{13}C{^{1}H} (100.67 MHz, $CDCl_3$) δ 158.1 (d, $J_{C\text{-}F}$ = 254.1 Hz), 156.2 (d, $J_{C\text{-}F}$ = 4.0 Hz), 137.0 (d, $J_{C\text{-}F}$ = 23.4 Hz), 124.01 (d, $J_{C\text{-}F}$ = 3.8 Hz), 123.0 (d, $J_{C\text{-}F}$ = 18.2 Hz), 59.3, 55.9, 53.8, 35.0. ^{19}F (376.7 MHz, $CDCl_3$): -

131.6 (t, J_{F-H} = 6 Hz) HRMS (ESI) (m/z): [M+Na] calcd for $C_{16}H_{19}F_2N_3NaO$ 330.1394, found 330.1389.

Compound 6a, 6 :

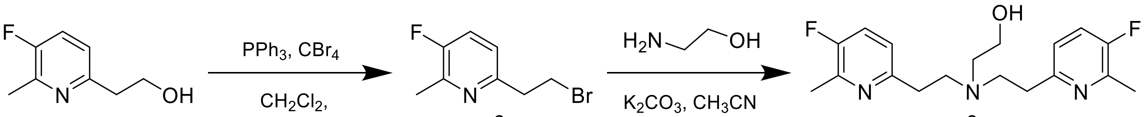

6a: To a solution of 2-(5-fluoro-6-methylpyridin-2yl)ethan-1-ol (250 mg, 1.61 mmol) in CH_2Cl_2 (5 mL), PPh_3 (1.06 g, 4.03 mmol) was added followed by CBr_4 (1.34 g, 4.03 mmol) at 0 °C. The reaction mixture was stirred at room temperature for 6 h, and completion of the reaction was confirmed by TLC. The solvent was removed under reduced pressure, and the resulting residue was purified by silica gel column chromatography (EtOAc/Petroleum ether, 5:95) to obtain compound **6a** as yellow oil (260 mg, 74%). LC/MS (ES, *m/z*) [M+H]: 219.

6: Compound **6a** (260 mg ,1.20 mmol) was added to a solution of 2-aminoetha-1-ol (32 mg, 523.87 μmol) in CH_3CN (3 mL) and stirred under N_2 for 5 min at room temperature followed by addition of potassium carbonate (217.20 mg, 1.57 μmol), then the reaction mixture was heated to 80 °C for 5 h. Completion of the reaction was confirmed by TLC and the solvent was evaporated. The crude product was dissolved in $CHCl_3$, washed with brine solution and extracted with $CHCl_3$ (2×20 mL), and the combined organic phase was dried over Na_2SO_4. Following filtration, the organic phase was concentrated and purified using a silica gel column chromatography (EtOAC/Petroleum ether 80:20) to obtain compound **6** as yellowish white solid (130 mg, 74%).^{1}H NMR (400.35 MHz, $CDCl_3$) δ 7.22 (t, J_{H-F} = 8.62 Hz, 2H), 6.86 (m, 2H), 3.63 (t, J = 4.6 Hz, 2H), 2.97 (t, J = 6.6 Hz, 4H), 2.86 (t, J = 6.6 Hz, 4H), 2.79 (t, J = 4.6 Hz, 2H), 2.54 (s, 6H). $^{13}C\{^1H\}$ (100.67 MHz, $CDCl_3$) δ 157.6, 155.1 (d, J_{C-F} = 4.7 Hz), 145.6 (d, J_{C-F} = 16.5 Hz), 122.3 (d, J_{C-F} = 19.2 Hz), 121.6 (d, J_{C-F} = 3.**5** Hz), 59.4, 56.1, 54.1, 35.0, 17.7. ^{19}F (376.7 MHz, $CDCl_3$): -130.2 (m). [M+H] calcd for $C_{18}H_{24}F_2N_3O$ 336.1887, found 336.1882.

C. ^{19}F-iCEST NMR experiments

All ^{19}F-iCEST NMR experiments were performed on a 9.4 T MHz AVANCEIII NMR spectrometer (Bruker, Germany), with the sample temperature stabilized at 310 K. A pre-saturation pulse (t_{sat}) with a length of 2 sec and a pulse strength 100 Hz were applied prior to the 90° RF pulse. The frequency of the B_1 was swept from Δω= +8 ppm to Δω= -8 ppm in 100 Hz = 0.27 ppm steps relative to the resonance of the free ^{19}F-iCEST sensor (set to 0 ppm for convenience). Each frequency offset (Δω +/ -) was acquired with eight scans, using a repetition time of 15 sec, resulting in total iCEST experiment time of ~2h 34 min.

D. Bloch-McConnell data fitting

To estimate the exchange rate (k_{ex}) between Zn^{2+}-bound and free ^{19}F-chelate, multi B_1 CEST experiments were perfumed with saturation powers ranging from 5 Hz to 150 Hz. The obtained data (z-spectra) were fitted using the Bloch-McConnell equations[1-3]. Both numerical and analytical simulations, which provided similar k_{ex} values, were performed using a custom-written script in MATLAB version 8.2.0.701 (The MathWorks, Natick, MA). The code for data-fitting can be found at http://www.cest-sources.org/doku.php?id=start.

E. Dissociation constant (K_d) measurements by ^{19}F NMR

Since the exchange between Zn^{2+}-bound and free **5** is slow in the NMR timescale, the K_d values can be evaluated from the ratio between the integrals of the two NMR signals obtained for the free and bound forms (Figure S2). Thus, the dissociation constant K_d was determined with the assistant of ^{19}F-NMR at 25 °C. To this end, ^{19}F-NMR spectra of aqueous solutions (in 100 mM Hhepes buffer, pH~7.4) of 1 mM of **5** in a presence of 2-5 mM of Zn^{2+} resulting in a variety of **5**:Zn^{2+} molar ratios (0.2:1, 0.3:1, 0.35:1, 0.4:1, 0.45:1, 0.5:1, see Table S1 and representative example in Figure S2) were recorded. Then, the ^{19}F-NMR signals of Zn^{2+}-bound ($\mathbf{5} \cdot Zn^{2+}$)

and free **5** were integrated and the obtained values were used for K_d calculations. In order to measure a true equilibrium constant, the concentrations were varied in order to obtain 20-80% of the bound form to determine the K_d value (K_d=5.5±0.6 $\times 10^{-3}$ M). See, Table S1 and Figure S2.

$$\mathbf{5} + Zn^{2+} \rightleftharpoons \mathbf{5} \cdot Zn^{2+}$$

$$K_d = \frac{[\mathbf{5}][Zn^{2+}]}{[\mathbf{5} \cdot Zn^{2+}]}$$

F. Crystallization procedure

Compound **3** + Zn Complex: Both Compound **3** (5 mg) and $Zn(ClO_4)_2 \cdot 6H_2O$ (6.6 mg) were dissolved in 50 µL MeOH separately. The obtained solutions were sonicated until they became clear, mixed at room temperature, and left for slow evaporation. After 14 days, a single crystal was obtained for X-ray crystallography analysis.

Compound **4** + Zn Complex: Both Compound **4** (5 mg) and $Zn(ClO_4)_2 \cdot 6H_2O$ (6 mg) were dissolved in 50 µL MeOH separately. The obtained solutions were sonicated until they became clear, mixed at room temperature, and left for slow evaporation. After 14 days, a single crystal suitable for X-ray crystallography analysis was obtained.

Compound **5** + Zn Complex: Both Compound **5** (5 mg) and $Zn(ClO_4)_2 \cdot 6H_2O$ (6 mg) were dissolved in 50 µL MeOH separately. The obtained solutions were sonicated until they became clear, mixed at room temperature, and left for slow evaporation. After five days, a single crystal suitable for X-ray crystallography analysis was obtained.

G. Cell Viability

Cell Titer-Blue assay: Chinese hamster ovary (CHO) cells (12 x 10^3 cells/mL) were cultured in 96-well microplates with Dulbecco's Modified Eagle Medium (DMEM) for 16 hr at 37 °C and 5% CO_2. Then, cells were washed with fresh cell medium

and treated with Compound **5**, which was dissolved in PBS over a range of concentrations (as noted in the graph). After 3.5 hr of incubation, the incubating medium was removed and cells were washed with PBS. Then, fresh DMEM medium (80 μL) was added and followed by the addition of a 20 μL of CellTiter-Blue® reagent (Promega) after which the fluorescence of each well ($\lambda_{ex}/\lambda_{em}$ of 573nm/584nm) was recorded using a dedicated plate reader. For each concentration of compound **5**, the average fluorescence value was calculated from six biological replicates. Cells treated with 50% DMSO were used as a positive control.

H. MRI experiments

Phantom Studies:

Phantom experiments were performed on a 9.4 T wide-bore MR scanner (Bruker Avance system) at 37 ^{o}C. A 25 mm, double-resonant ($^{1}H/^{19}F$) radiofrequency (RF) coil was used. Seven 5 mm NMR tubes containing 7 mM of compound **5** without the addition of cations (blank) or with 100 μM of either of the cations Ca^{2+}, Cu^{2+}, Mg^{2+}, Na^{+}, K^{+}, and Zn^{2+} in 100 mM Hepes buffer (pH ~ 7.4) were placed in a larger tube containing a 4% gelatin (w/w) solution in water. ^{1}H MRI: A RARE sequence was used to acquire ^{1}H MRI images with the following parameters: TR/TE=3000/42.4 ms; FOV=3.2 x 3.2 cm; matrix size=128 x 128; RARE factor=8; averages (NA=2). ^{19}F-iCEST MRI: A modified RAREst sequence at ^{19}F-MRI was used to perform the ^{19}F-iCESTMRI experiments with the following parameters: TR/TE=6000/11 ms; RARE factor=16; 8 mm slice; FOV=3.2 × 3.2 cm; matrix size=32 × 32; NA=100, and a saturation pulse of B_1= 2.5 μT for 2 s. The frequency of B_1 was swept from $\Delta\omega$= +8 ppm to $\Delta\omega$= -8 ppm in 150 Hz steps relative to the resonance of the **5** (set to 0 ppm).

***In vivo* MRI:**

Male C57BL/6 mice were purchased from Envigo (Israel). All the animal experiments were performed in accordance with the IACUC guidelines and regulations of the Weizmann Institute of Science. All animals survived the in vivo experiments with no observable undesired effects.

The following coordinates were used for intracranial implementation of the probe delivering cannula: For delivering **5** to the CA3 region of the hippocampus the cannula was located at the following coordinates (based on the mouse brain atlas): 2.42 mm (ML), 2.15 mm (AP), and 2.00 mm (DV), relative to bregma. For delivering **5** to the thalamus (TH) region the cannula was located at the following coordinates (based on the mouse brain atlas): 2.42 mm (ML), 2.15 mm (AP), and 3.50 mm (DV), relative to bregma. After successful cannula implantation at the location of interest in the animal brain, the animal was transferred to the Bruker BioSpec 15.2 Tesla AVANCE III HD imaging spectrometer equipped with a dual $^{1}H/^{19}F$, 23 mm RF coil. Compound **5** (10 mM at PBS) was the continuously infused (infusion rate was set to 0.25 μL/min) and ^{1}H-MRI followed by ^{19}F-iCEST data sets were aqcuired. ^{1}H MRI: A RARE sequence was used to acquire the ^{1}H MRI images with the following parameters: TR/TE=5000/34.7 ms; FOV=2.2 x 2.2 cm; matrix size=128 x 128; RARE factor=16, NA=2. Then, 90 min from starting the infusion of **5**, a ^{19}F-NMR experiment was performed from which the frequency offset of the delivered **5** was determined as the center frequency offset (O_1) and ^{19}F-iCEST MRI data sets were acquired with the following parameters: A modified CEST-RAREst sequence, TR/TE=3000/10.9 ms; RARE factor=16; 8 mm slice; FOV=2.2 × 2.2 cm; matrix size=32 × 32; NA=400; and a saturation pulse B_1= 2 μT for 1.5 s. Four data sets were acquired with the parameters mentioned above with two at which B_1 was applied “on resonance” (Δω =+3.2 ppm) and another two at which B_1 was applied “off resonance” (Δω =-3.2 ppm). These data sets were acquired in alternate order and were averaged out separately. To obtain the ^{19}F-iCEST MRI contrast, averaged “on resonance” images were subtracted from averaged “off resonance” images. One set of experiments was applied on a group of mice (N=7) implanted

with a cannula at the CA3 region where Δω was applied at either +18 ppm or -18 ppm and that data was processed in a similar way to the described above.

I. Supporting Information Figures:

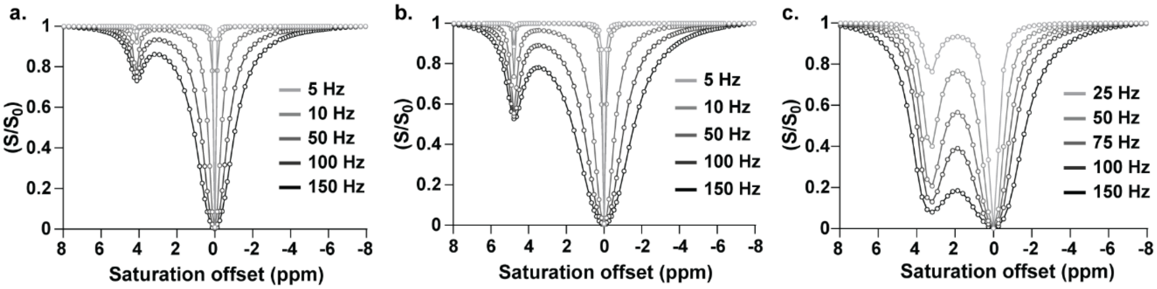

Figure S1: Experimental z-spectra of multi power ^{19}F-iCEST experiments for compound 3,4,5 with Zn^{2+}. a) 3 mM of compound **3** and 120 μM of Zn^{2+}. b) 3 mM of compound **4** and 30 μM of Zn^{2+}. c) 3 mM of compound **5** and 40 μM of Zn^{2+}. All the solutions were prepared in 100 mM Hepes buffer (pH ~ 7.4) and ^{19}F-iCEST experiments was performed on 9.4 T NMR at 37 °C.

Table S1: Dissociation constant (K_d) determination by ^{19}F-NMR. K_d [**5**-Zn^{2+}]= 5.5±0.6 ×10^{-3} M.

Prepared solution (ratio of Zn^{2+}:**5**)	Compound **5** [mM]	Zn^{2+} [mM]	[**5**-Zn]/[**5**] (NMR integrals)	K_d [× 10^{-3}M]
1:0.5	1	2	0.32	5.506
1:0.45	1	2.222	0.314	6.307
1:0.4	1	2.5	0.424	5.187
1:0.35	1	2.85	0.452	5.6
1:0.3	1	3.3333	0.655	4.47
1:0.2	1	5	0.759	6.00

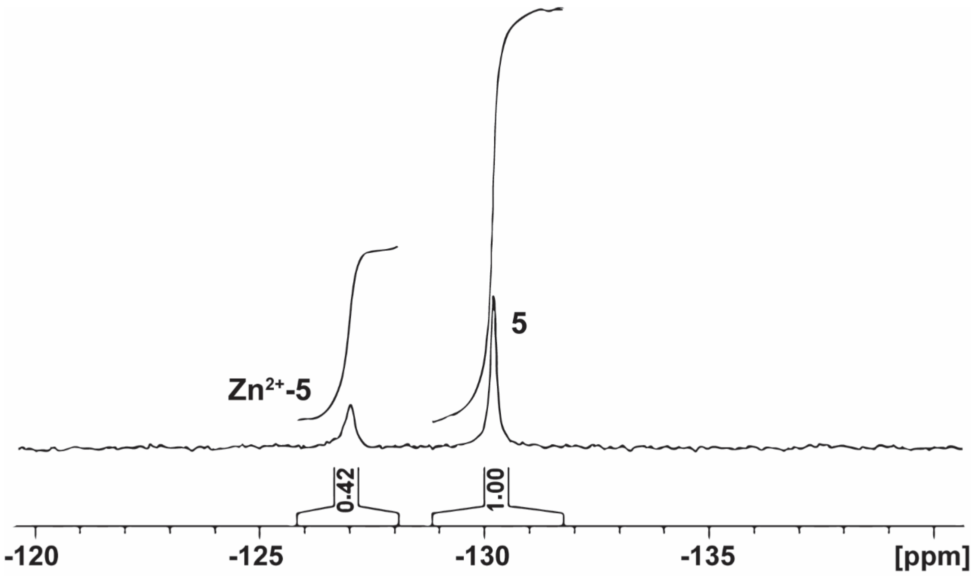

Figure S2: ^{19}F-NMR for K_d determination: Representative ^{19}F-NMR spectrum of solution prepared with **5**:Zn^{2+} ration of 1:0.4 (in 100 mM Hepes buffer, pH ~ 7.4 at 25 °C on 11.7 T NMR spectrometer). The noted integration values were used to calculate the dissociation constant (K_d).

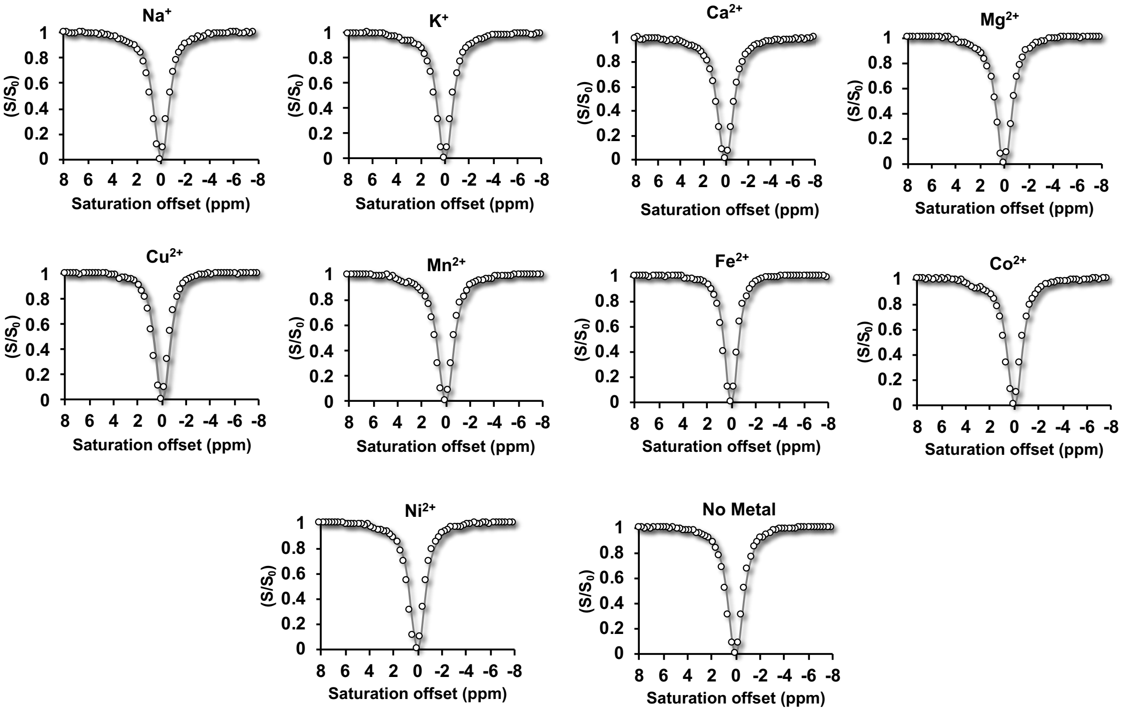

Figure S3: ^{19}F-iCEST spectra of 3 mM of compound **5** in presence of 30 μM of (Na^{+}, K^{+}, Mg^{2+}, Ca^{2+}, Mn^{2+}, Fe^{2+}, Co^{2+}, Ni^{2+}, Cu^{2+}, no metal) in 100 mM Hepes buffer (pH 7.2) at 37 °C.

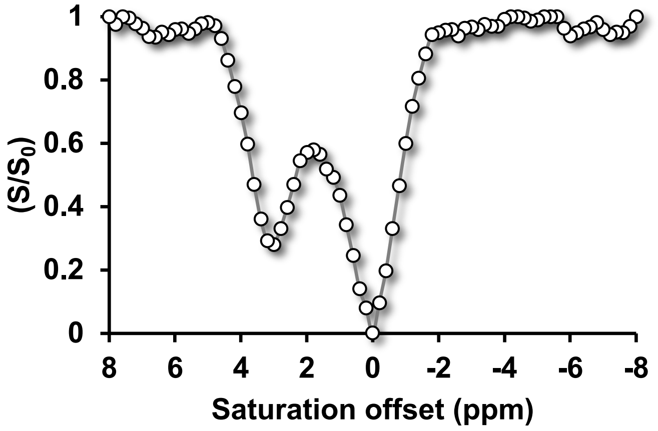

Figure S4: ^{19}F-iCEST MRI Z-spectrum of phantom tube containing Zn^{2+}: 7 mM of compound **5** and 100 μM of Zn^{2+} was dissolved in 100 mM Hepes buffer (pH=7.4) and ^{19}F-iCEST MRI experiment was performed on 9.4 T wide-bore MR scanner.

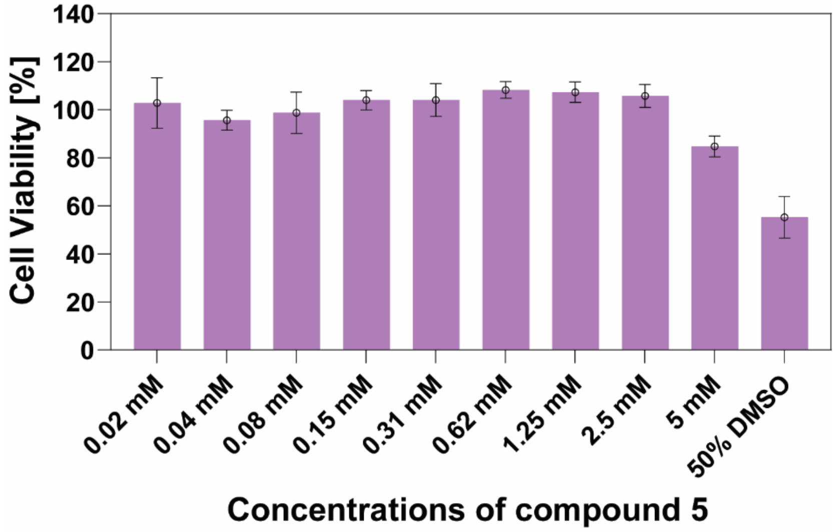

Figure S5: Cell Titer-Blue assay: Concentration-response bar-graph of cell Titer-Blue assay performed on CHO cells representing cell viability as a function of the concentration of compound **5** in the medium. Cells were incubated with the shown concentration for 3.5 h at 37 ºC. Treatment with 50% DMSO was used as a positive control. The data is shown as the percentile of live cells (relative to untreated cells) and expressed as mean ± SD of six repetitions.

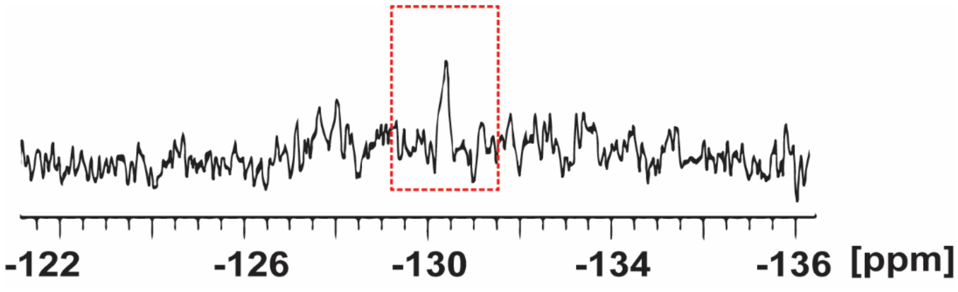

Figure S6: ^{19}F NMR of cells: Cells were incubated with 2 mM of compound **5** for 3.5 h and ^{19}F NMR was performed on 9.4 T NMR to check whether compound **5** enters the cells or not.

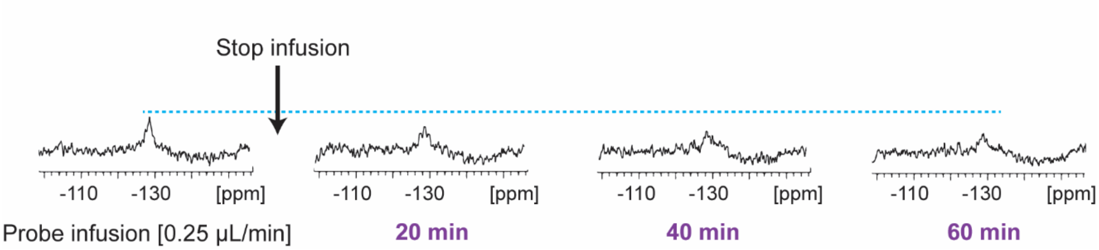

Figure S7: Washout studies of **5** *in vivo*: ^{19}F-NMR spectrum (left) obtained from a mouse brain following 90 min of **5** infusion (10 mM in PBS, 0.25 µL/min rate). The black arrow shows the time at which the infusion of **5** was stopped. Then a set of ^{19}F-NMR spectra were acquired 20, 40 and 60 min after stopping the delivery of **5** representing the probe washout from the tissue (^{19}F-signal drops over time).

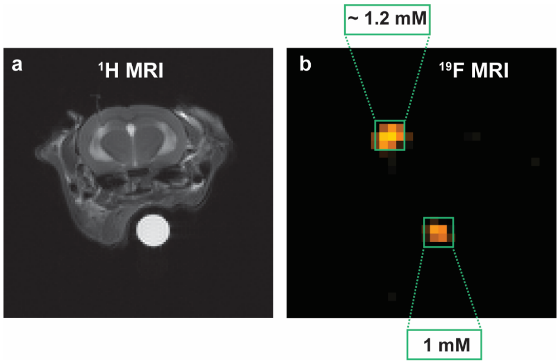

Figure S8: Estimation of the concentration of 5 in the mouse brain: Ninety minutes after starting the infusion of **5** (10 mM in PBS, 0.25 µL/min rate) its concentration was estimated using the ^{19}F-MRI signal of a reference tube that contained 1 mM of **5** in PBS. a. ^{1}H MRI (matrix size 128 $\times$ 128), b. ^{19}F MRI (matrix size 32 $\times$ 32). From the obtained ^{19}F-MRI the concentration of **5** in the brain was determined to be 1.2 mM.

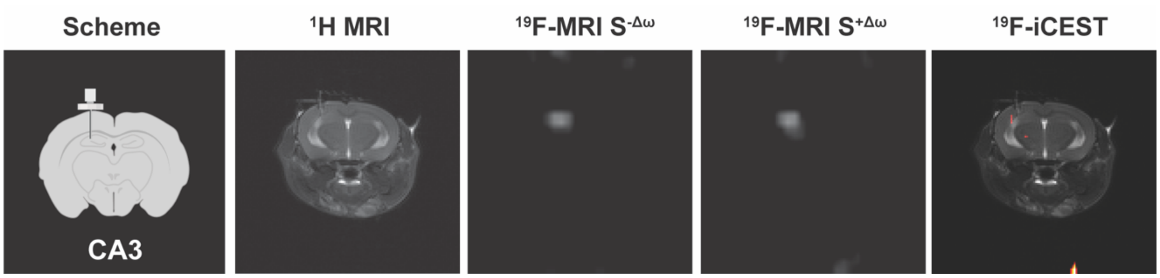

Figure S9: ***In vivo*** **^{19}F-iCEST maps in live animal brain at 18 ppm**. Continuous delivery of 10 mM compound **5** to CA3 in hippocampal region of brain *via* implanted cannula, ^{1}H MRI, ^{19}F-iCEST at $\Delta\omega$ = -18 ppm, ^{19}F-iCEST at $\Delta\omega$ = +18 ppm, ^{19}F-iCEST contrast overlaid on ^{1}H MRI. The infusion rate of **5** was set to 0.25 μL/min and iCEST data acquisition started 90 min from starting the infusion of **5**.

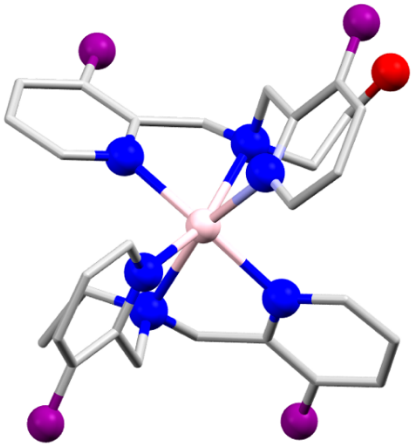

Figure S10: Crystal structure of compound **2** + **Zn^{2+}**.

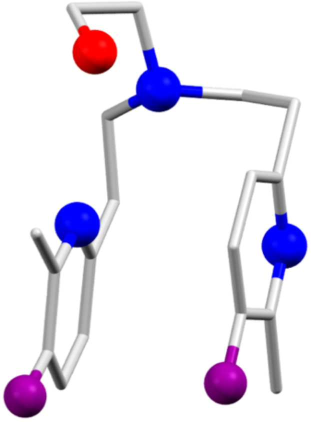

Figure S11: Crystal structure of compound **6**.

J. NMR (^{1}H, ^{13}C, ^{19}F) & HRMS Spectra

Compound 1 (^{1}H, ^{13}C, ^{19}F, HRMS)

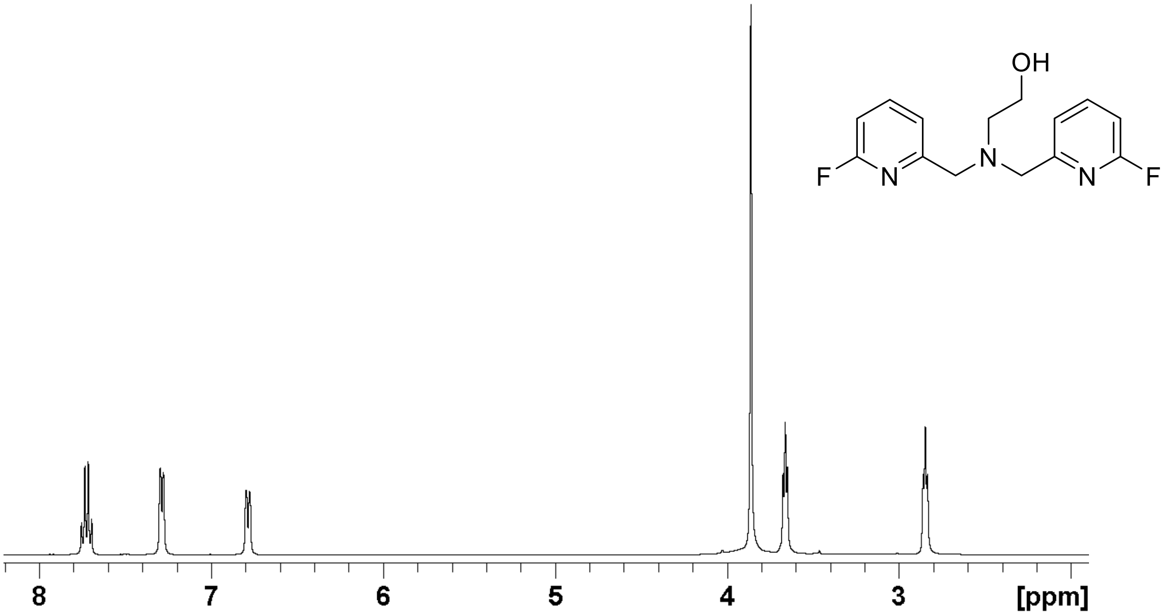

Figure S12: ^{1}H NMR (400.35 MHz, $CDCl_3$, 25 °C) spectrum of compound **1**.

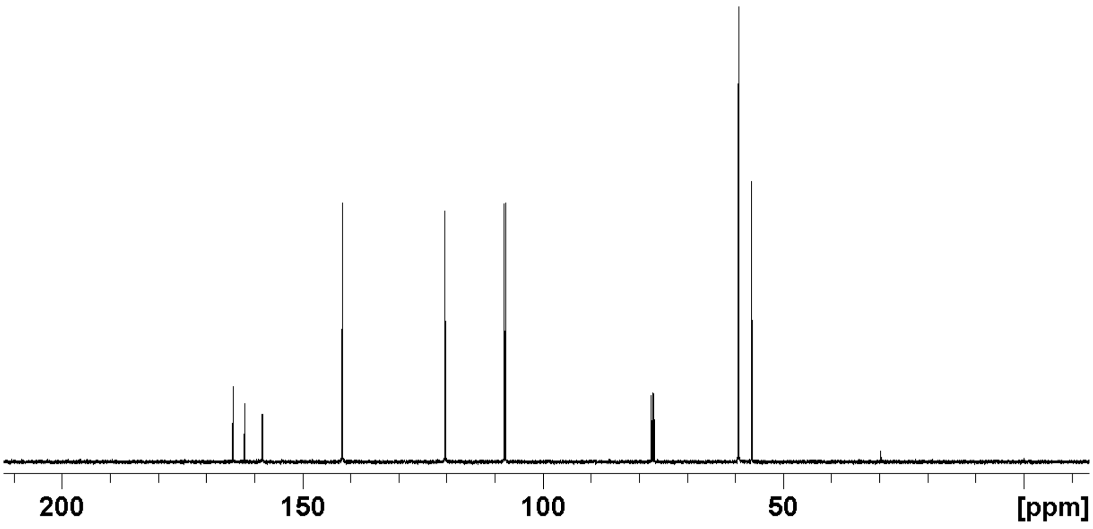

Figure S13: $^{13}C\{^1H\}$ NMR (100.67 MHz, $CDCl_3$, 25 °C) spectrum of compound **1**.

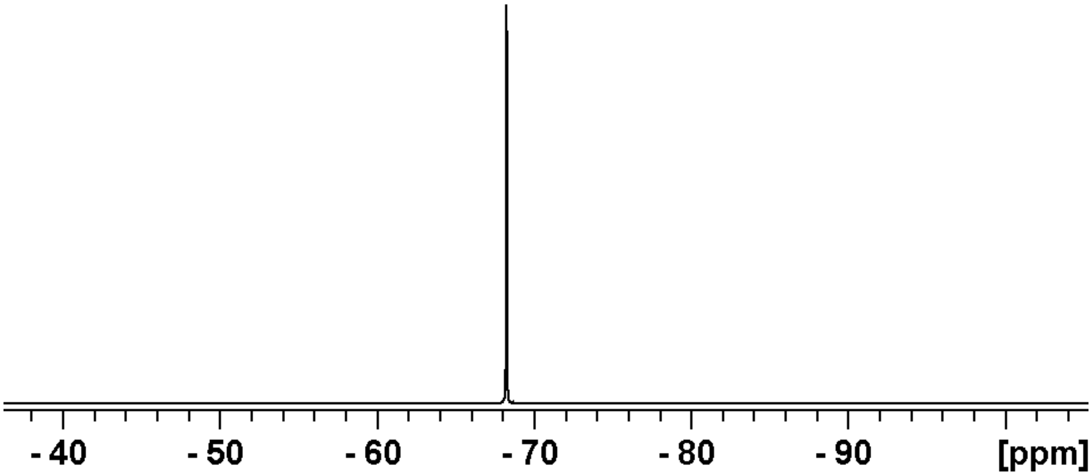

Figure S14: ^{19}F NMR (376.7 MHz, $CDCl_3$, 25 °C) spectrum of compound **1**.

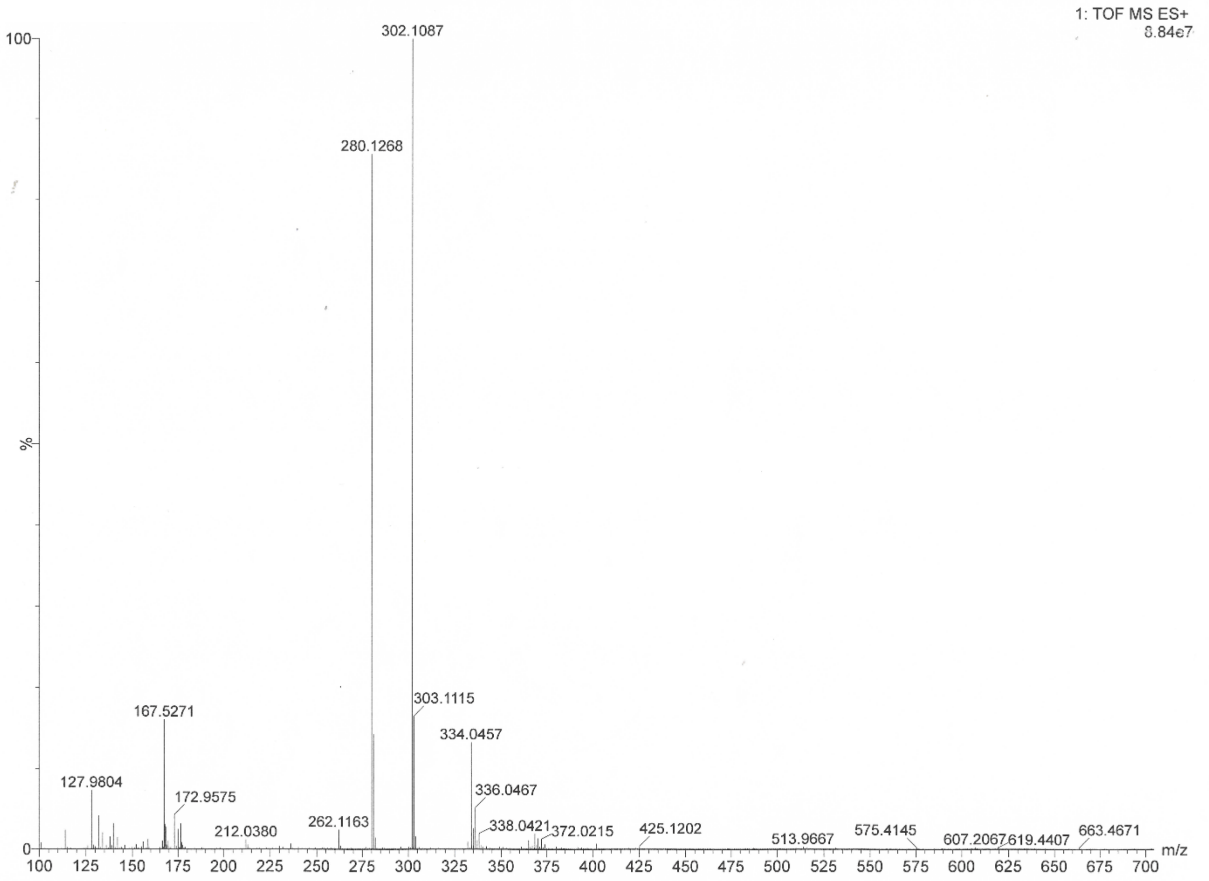

Figure S15: HRMS (ESI) spectrum of compound **1**.

Compound 2 (^{1}H, ^{13}C, ^{19}F, HRMS)

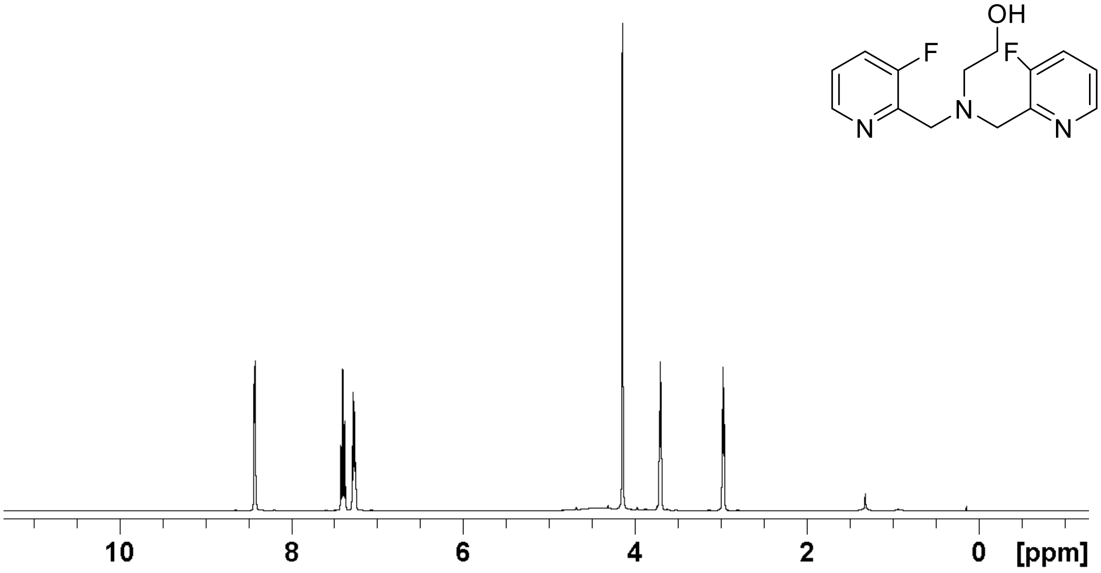

Figure S16: ^{1}H NMR (400.35 MHz, $CDCl_3$, 25 °C) spectrum of compound **2**.

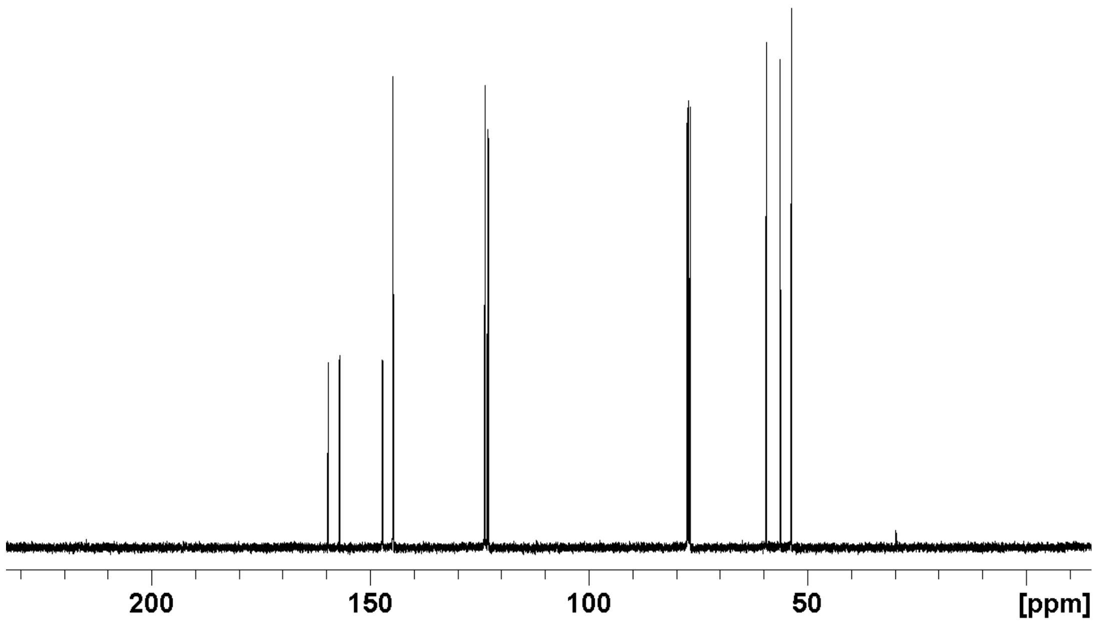

Figure S17: $^{13}C\{^{1}H\}$ NMR (100.67 MHz, $CDCl_3$, 25 °C) spectrum of compound **2**.

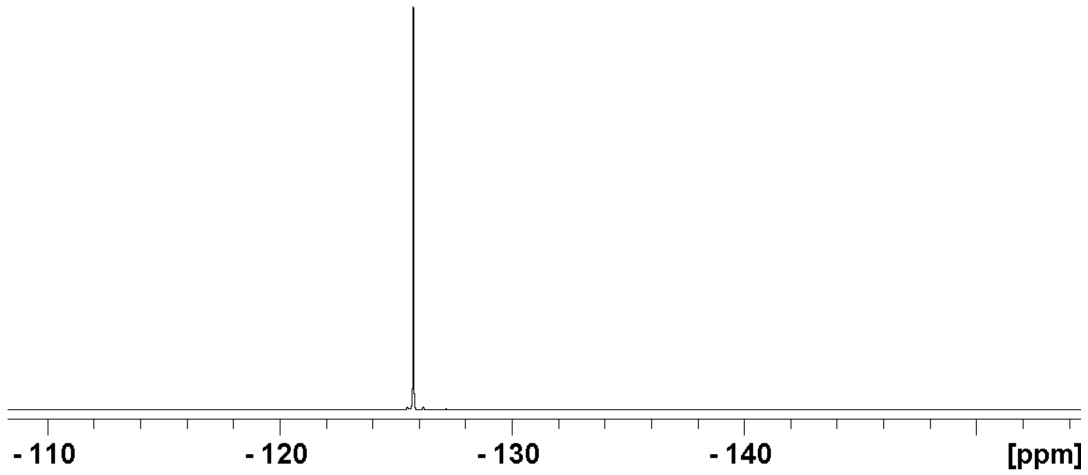

Figure S18: ^{19}F NMR (376.7 MHz, $CDCl_3$, 25 °C) spectrum of compound **2**.

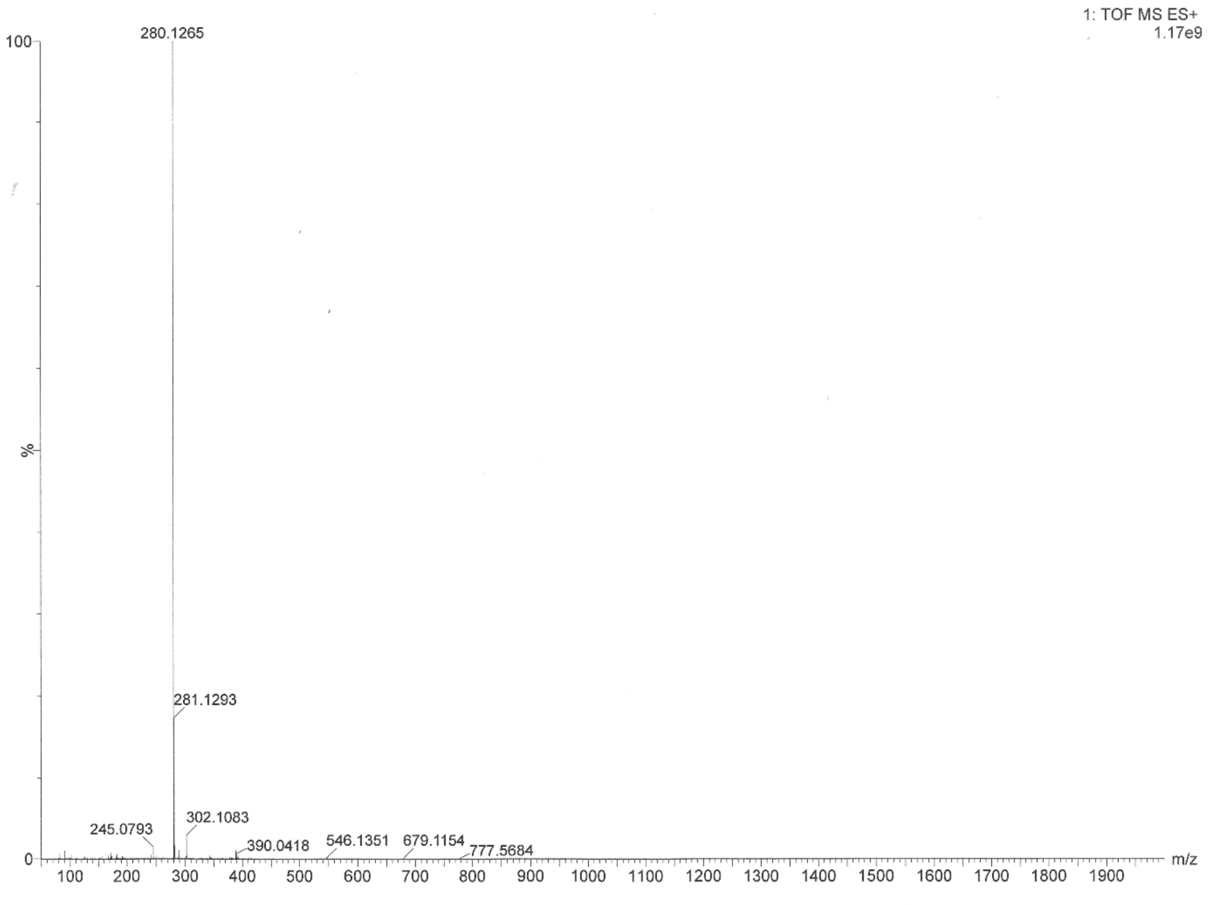

Figure S19: HRMS (ESI) spectrum of compound **2**.

Compound 3 (^{1}H, ^{13}C, ^{19}F, HRMS)

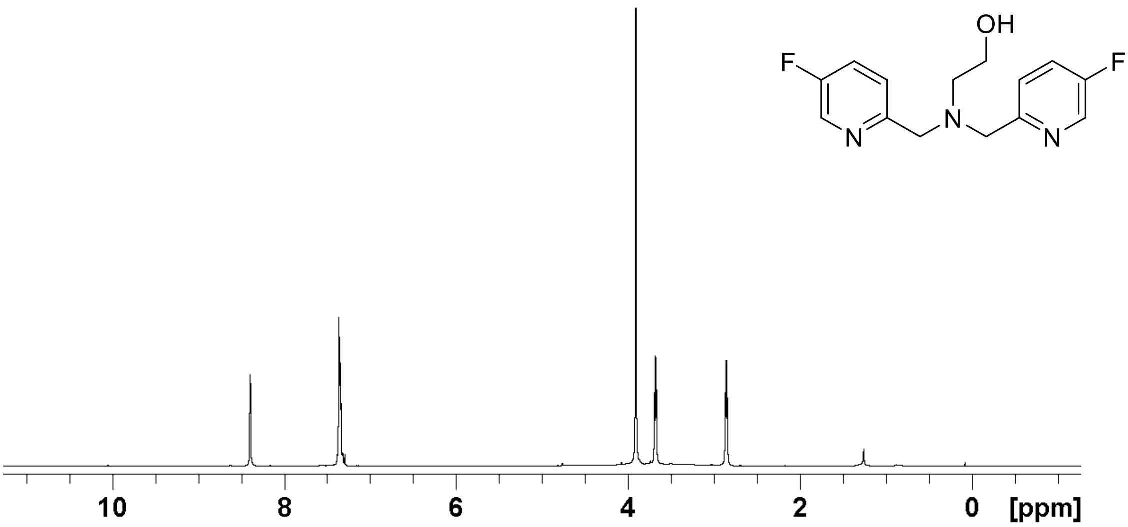

Figure S20: ^{1}H NMR (400.35 MHz, $CDCl_3$, 25 °C) spectrum of compound **3**.

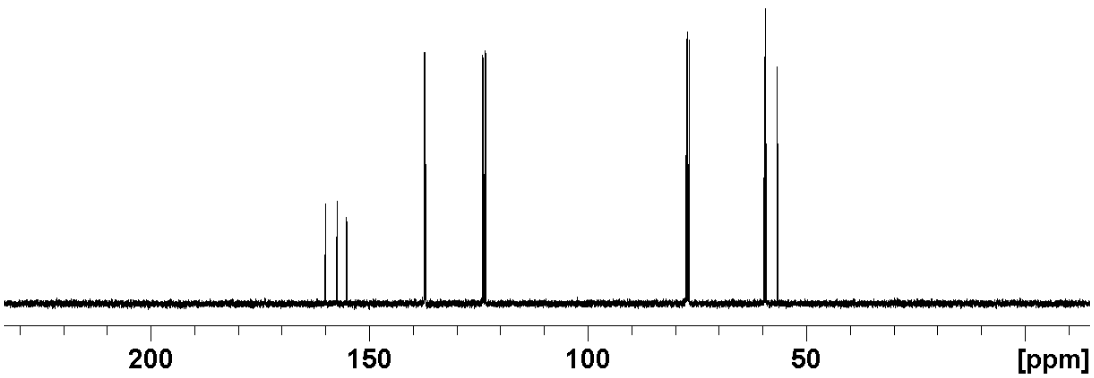

Figure S21: $^{13}C\{^{1}H\}$ NMR (100.67 MHz, $CDCl_3$, 25 °C) spectrum of compound **3**.

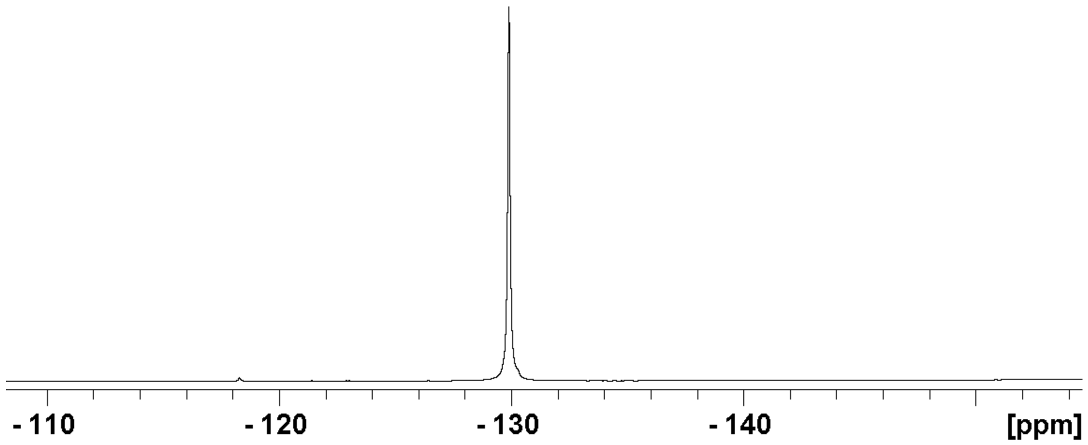

Figure S22: ^{19}F NMR (376.7 MHz, $CDCl_3$, 25 °C) spectrum of compound **3**.

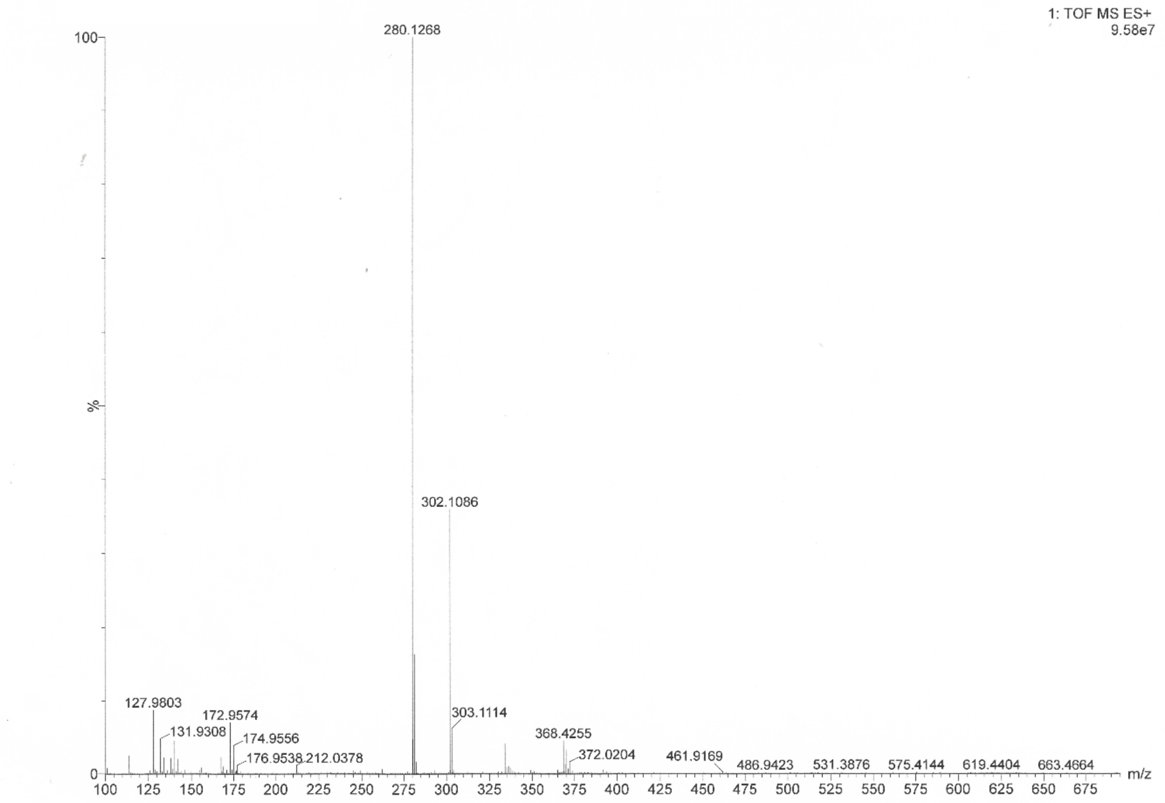

Figure S23: HRMS (ESI) spectrum of compound **3**.

Compound 4 (^{1}H, ^{13}C, ^{19}F, HRMS)

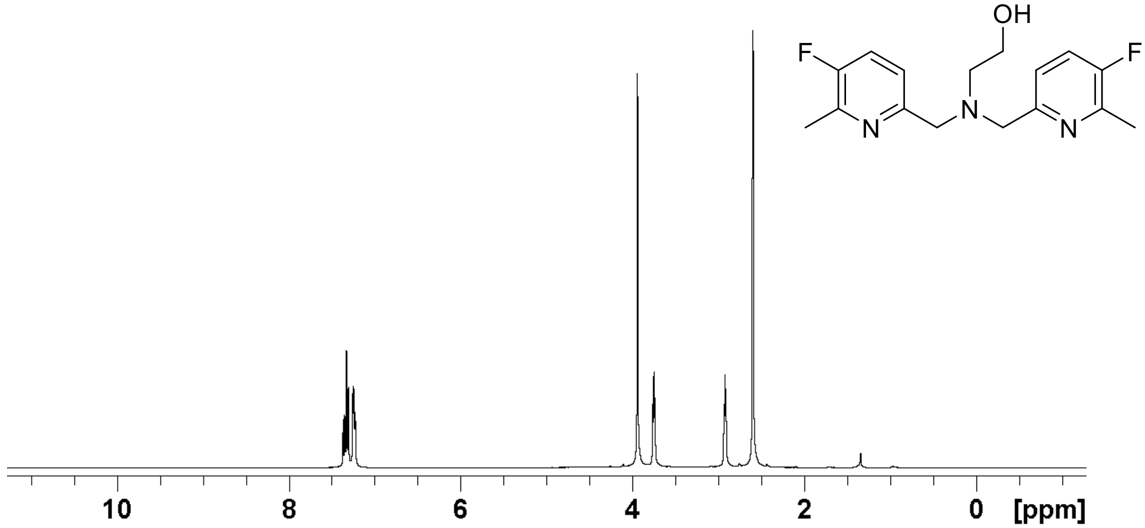

Figure S24: ^{1}H NMR (400.35 MHz, $CDCl_3$, 25 °C) spectrum of compound **4**.

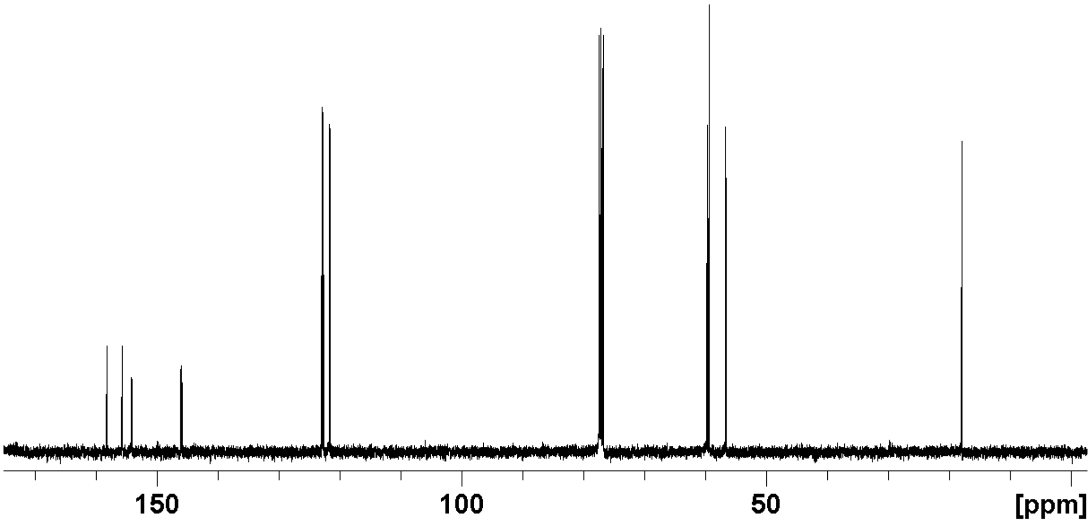

Figure S25: $^{13}C\{^{1}H\}$ NMR (100.67 MHz, $CDCl_3$, 25 °C) spectrum of compound **4**.

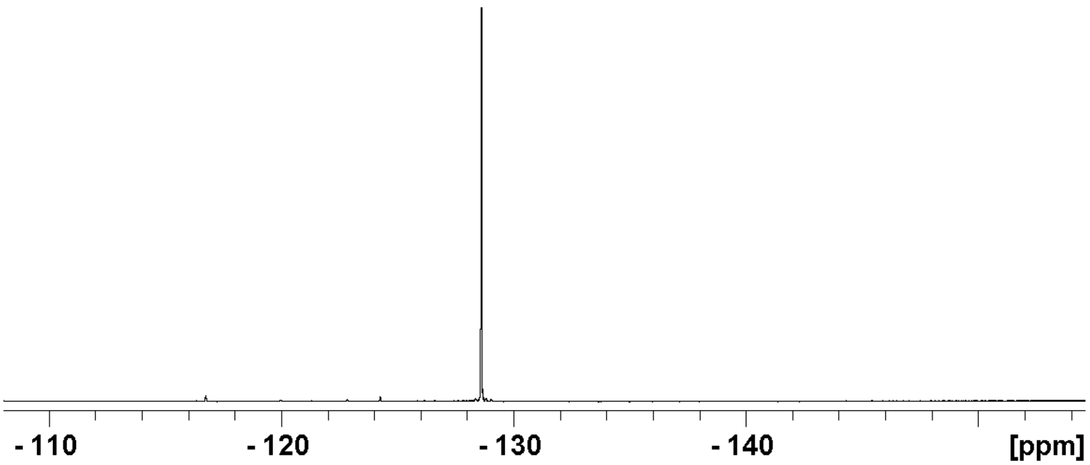

Figure S26: ^{19}F NMR (376.7 MHz, $CDCl_3$, 25 °C) spectrum of compound **4**.

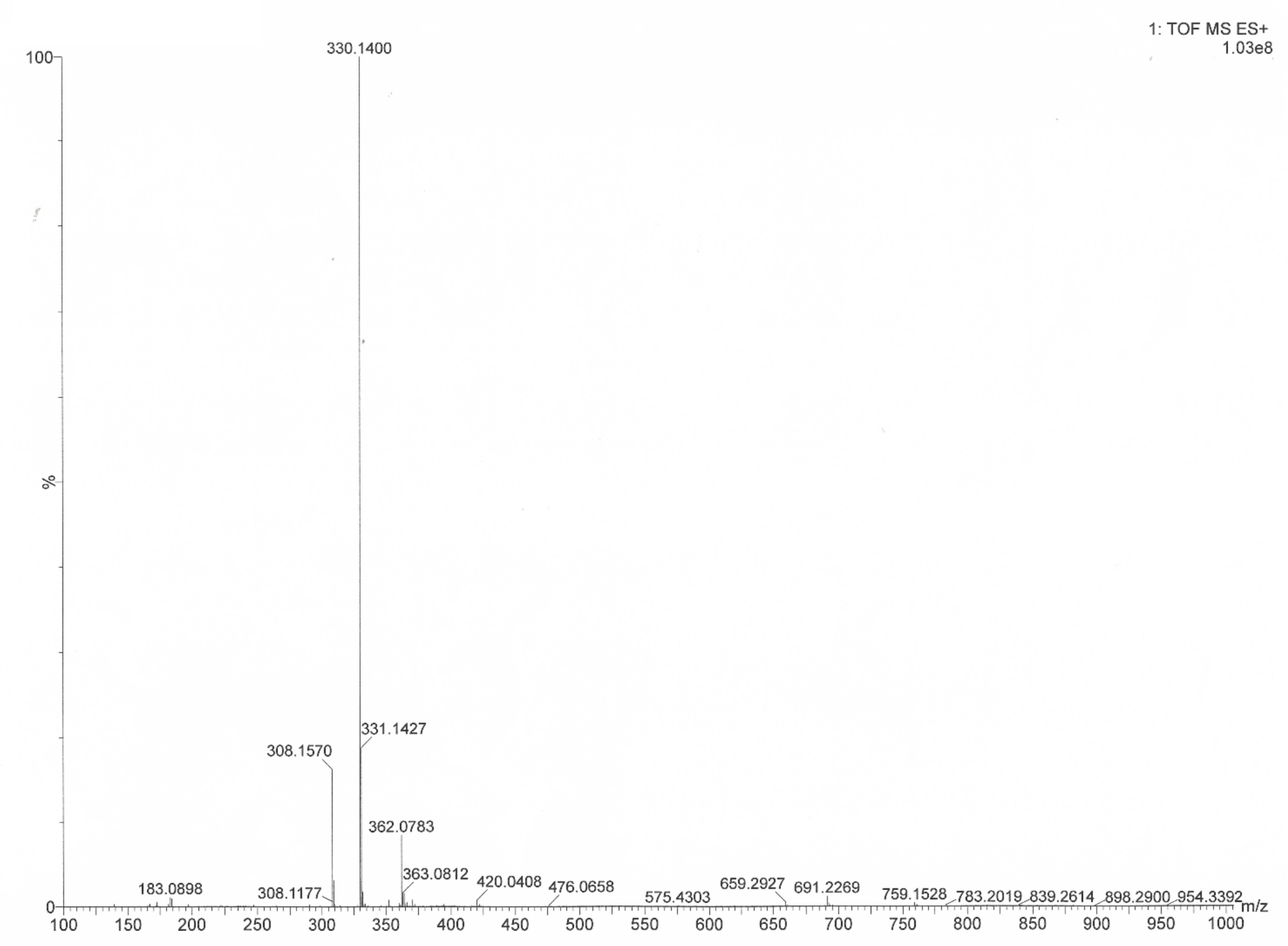

Figure S27: HRMS (ESI) spectrum of compound **4**.

Compound 5 (^{1}H, ^{13}C, ^{19}F, HRMS)

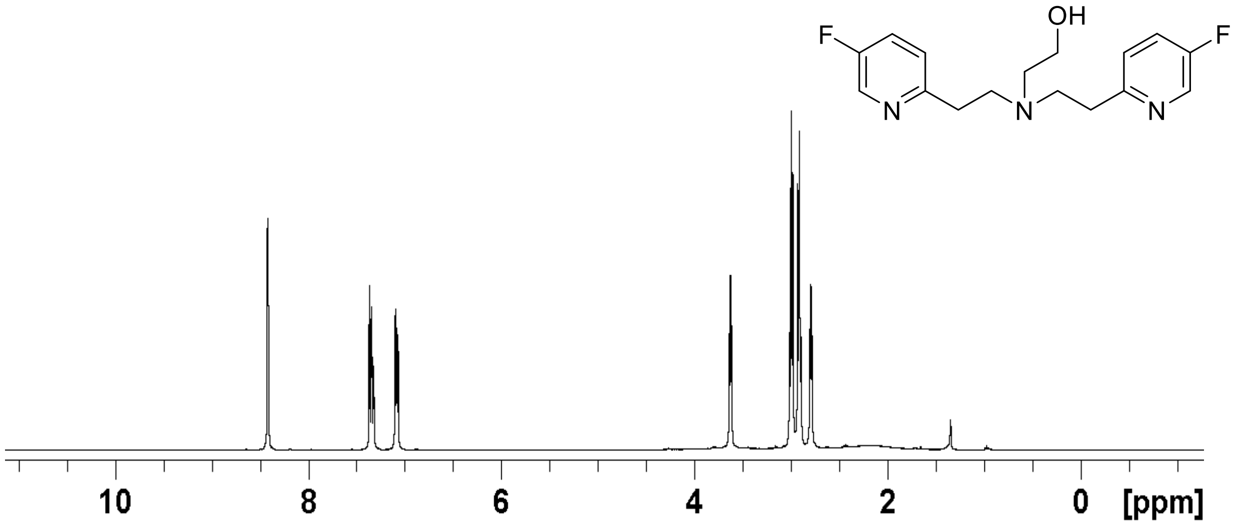

Figure S28: ^{1}H NMR (400.35 MHz, $CDCl_3$, 25 °C) spectrum of compound **5**.

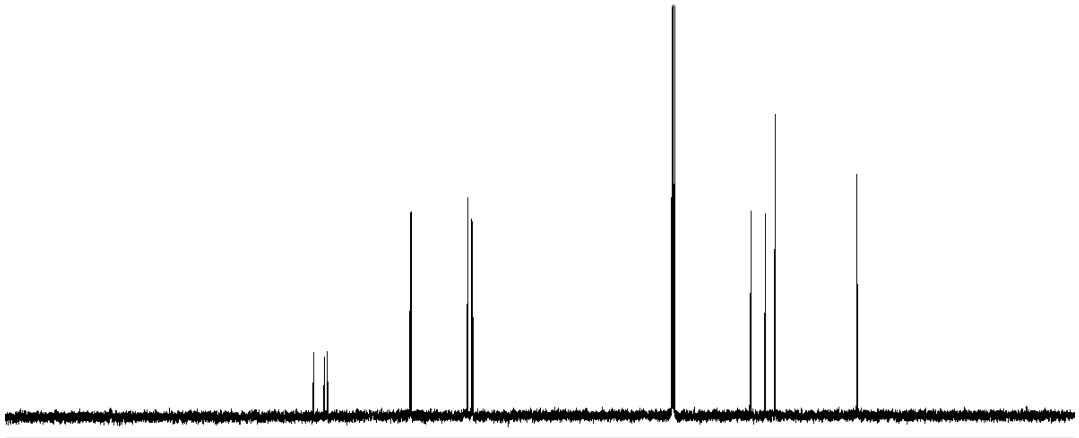

Figure S29: $^{13}C\{^{1}H\}$ NMR (100.67 MHz, $CDCl_3$, 25 °C) spectrum of compound **5**.

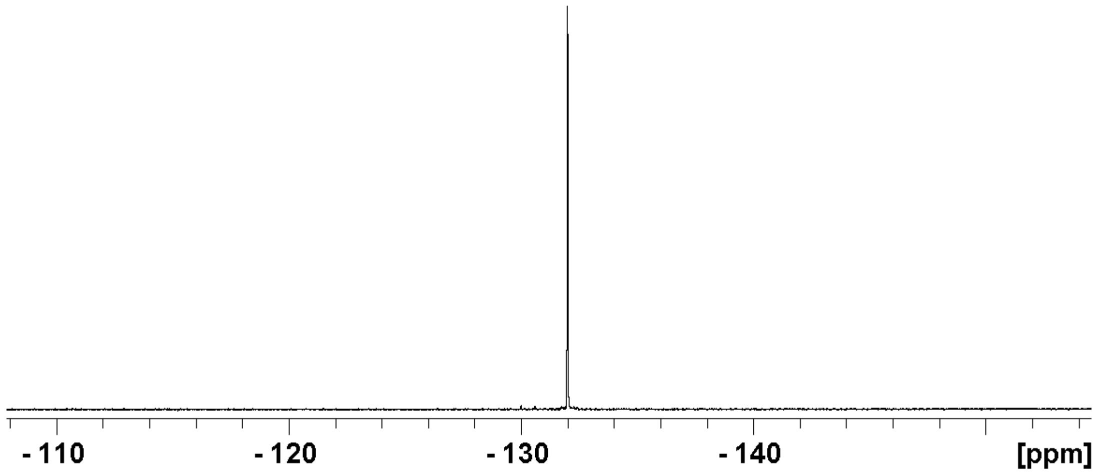

Figure S30: ^{19}F NMR (376.7 MHz, $CDCl_3$, 25 °C) spectrum of compound **5**.

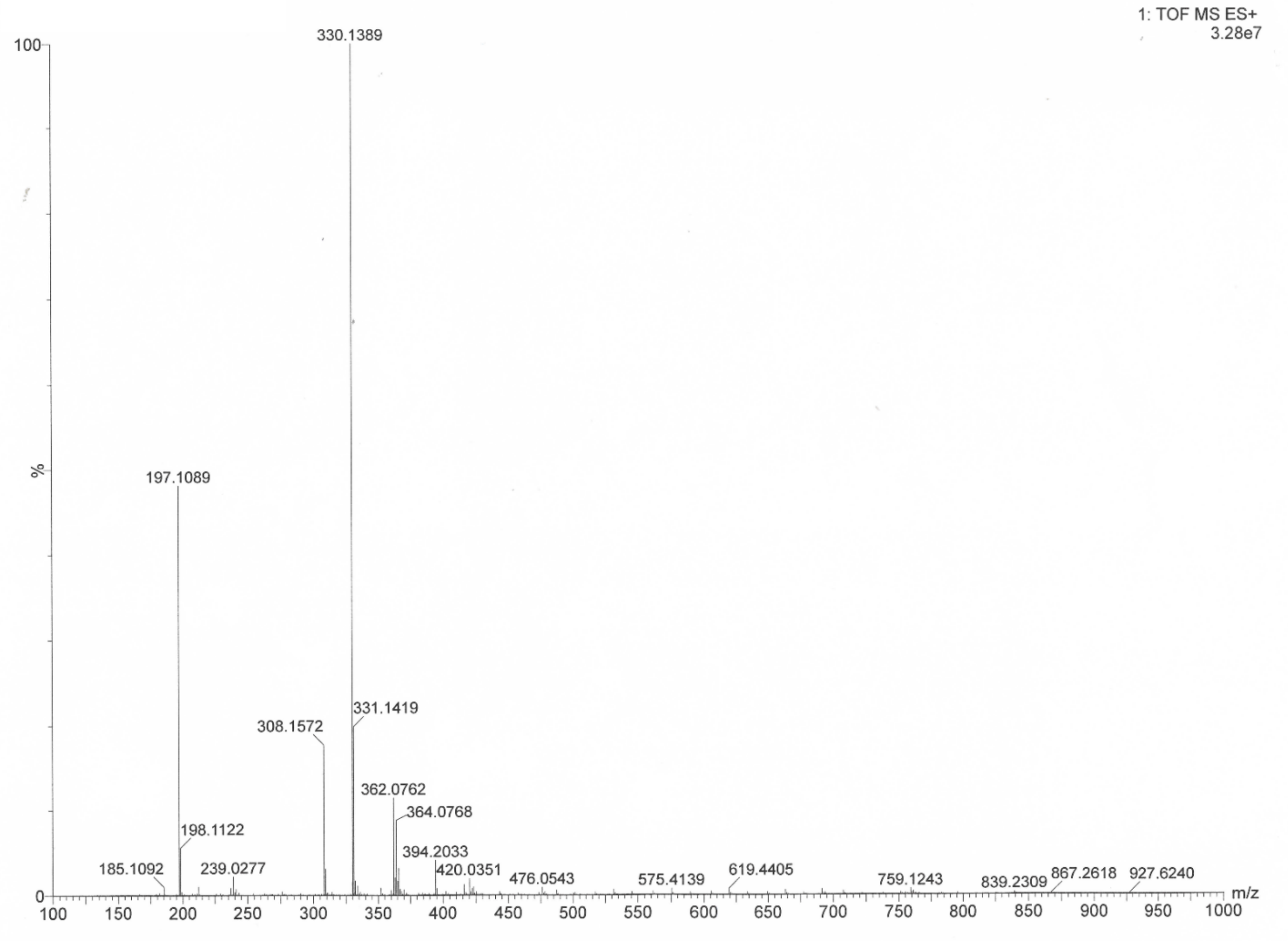

Figure S31: HRMS (ESI) spectrum of compound **5**.

Compound 6 (^{1}H, ^{13}C, ^{19}F, HRMS)

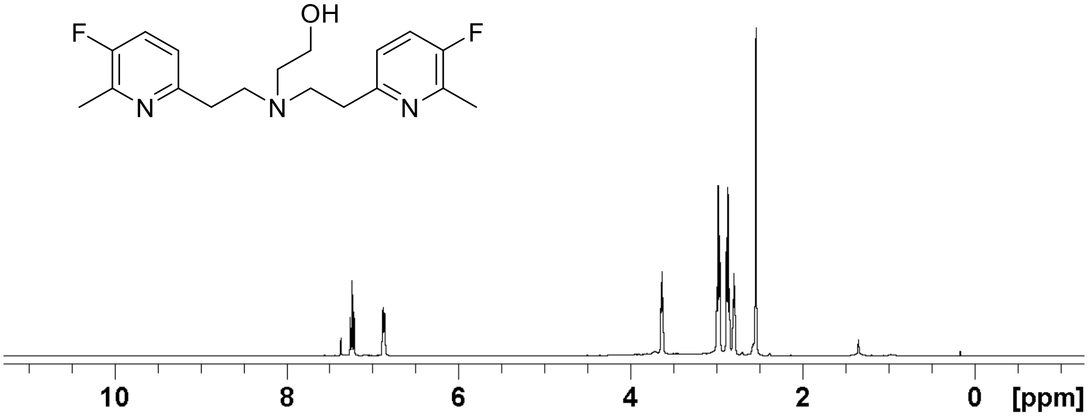

Figure S32: ^{1}H NMR (400.35 MHz, $CDCl_3$, 25 °C) spectrum of compound **6**.

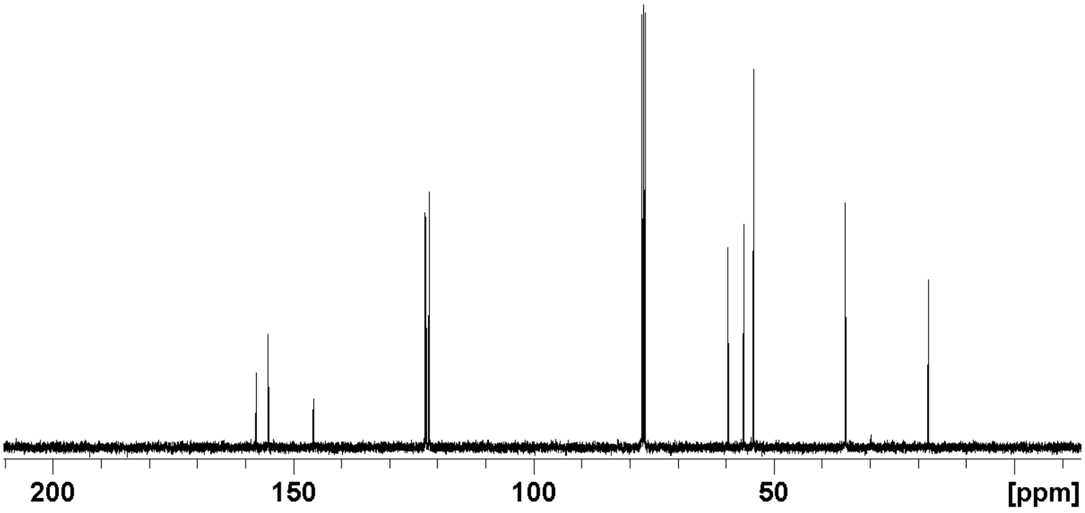

Figure S33: ^{13}C{^{1}H} NMR (100.67 MHz, $CDCl_3$, 25 °C)spectrum of compound **6**.

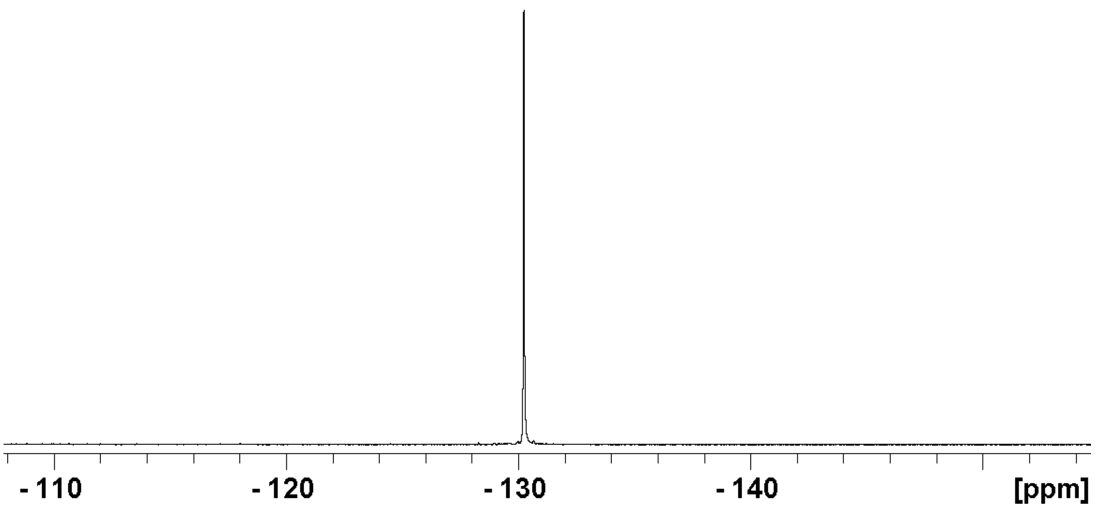

Figure S34: ^{19}F NMR (376.7 MHz, $CDCl_3$, 25 °C) spectrum of compound **6**.

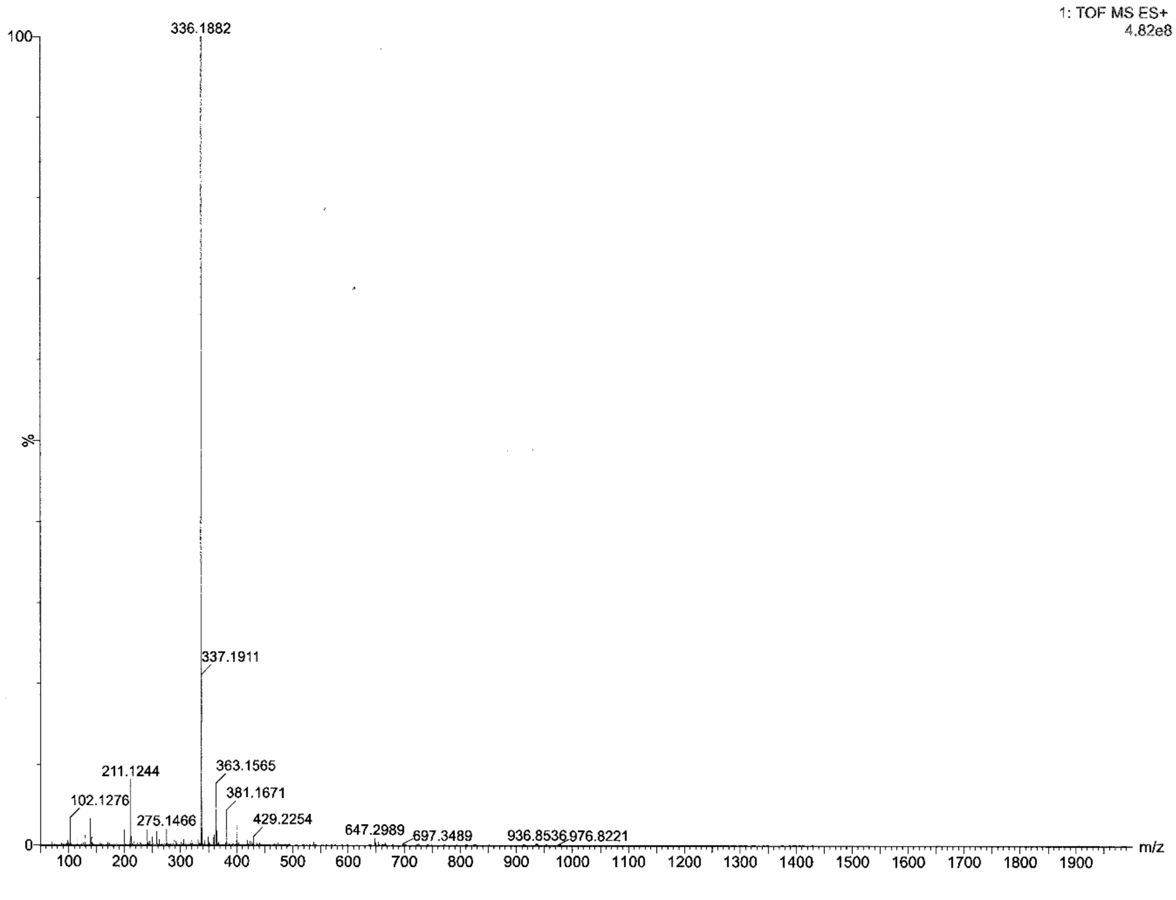

Figure S35: HRMS (ESI) spectrum of compound **6**.

K. Crystallographic data

Single crystals were obtained by slow evaporation of MeOH. Data were collected after immersion of a crystal in paratone oil and immediate flash cooling under liquid nitrogen stream. Single crystal X-ray diffraction data were collected on a sealed tube Rigaku Synergy-S dual source diffractometer equipped with Dectris Pilatus3 R CdTe 300K detector and microfocus, using Mo-Kα radiation (0.71073 Å). Data collection was performed under LN at 100 K. Data were processed with CrysAlis^{PRO} and the structures were solved by direct methods using the SHELXT[4]. Refinement was performed based on F^2 with SHELXL[5] and OLEX2[6]. Hydrogen atoms were assigned isotropic in riding mode. The twin structures were both two component twin structure with rotation of about 180°. Twin structures were first solved and refined against HKL4 reflection file and then further refined against the HKL5 reflections file. Crystallographic data and refinement parameters are shown as ORTEP (Oak Ridge Thermal Ellipsoid Plot) presentations in Figures S33-S37 and summarized in Supplementary Table S2.

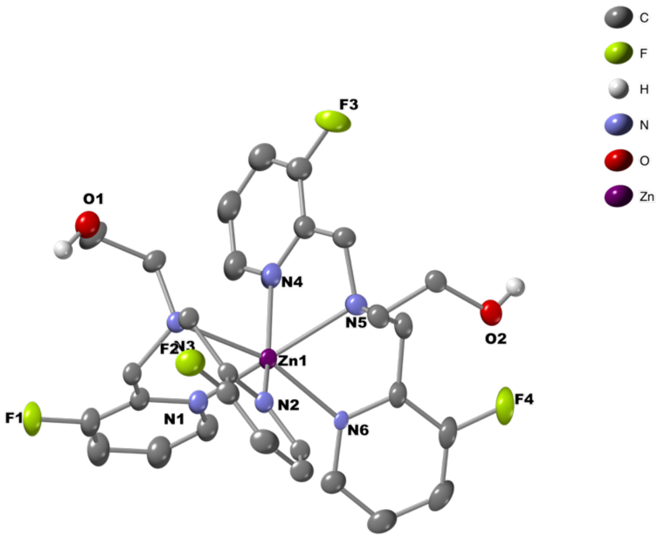

Figure S36. **ORTEP representation of the crystal structure of 2 + Zn**. Atoms are presented as thermal ellipsoids with 50% probability. Perchlorate counter ions and hydrogens are omitted for clarity. The hydroxy hydrogens are presented as spheres.

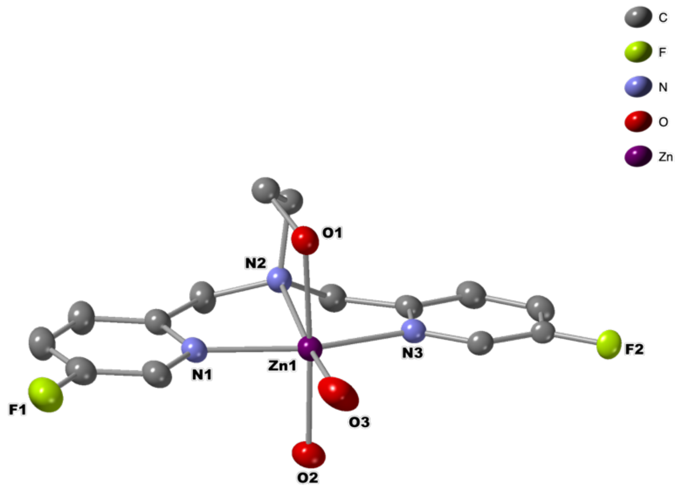

Figure S37. **ORTEP representation of the crystal structure of 3 + Zn**. Atoms are presented as thermal ellipsoids with 50% probability. Perchlorate counter ions and hydrogens are omitted for clarity.

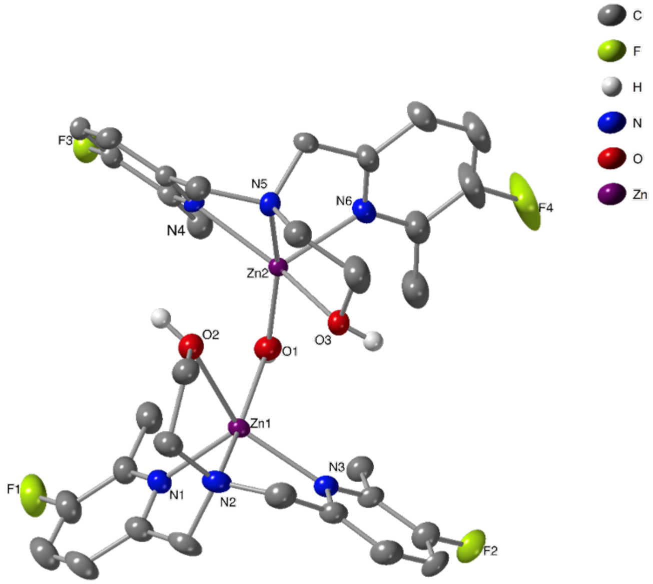

Figure S38. **ORTEP representation of the crystal structure of 4 + Zn**. Atoms are presented as thermal ellipsoids with 50% probability. Perchlorate counter ions and most of the hydrogens are omitted for clarity. Hydroxy hydrogens are presented as spheres.

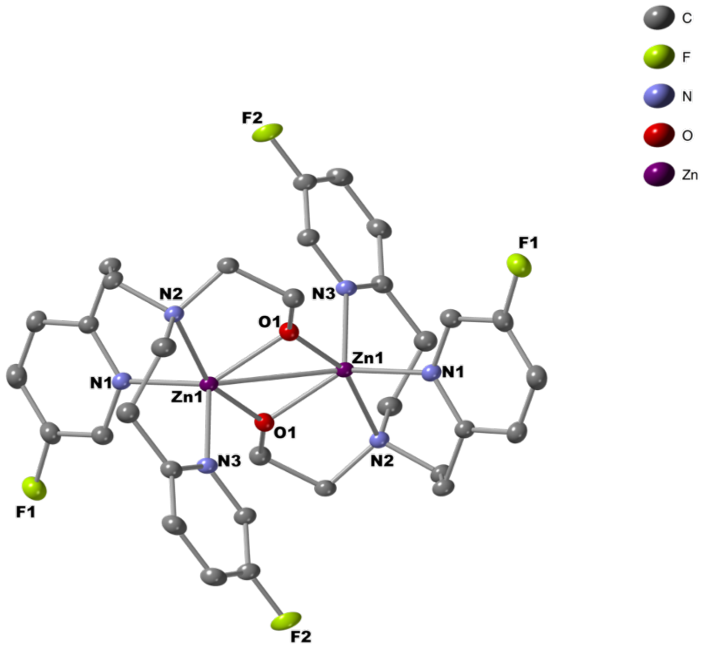

Figure S39. **ORTEP representation of the crystal structure of 5 + Zn**. Atoms are presented as thermal ellipsoids with 50% probability. Perchlorate counter ions and hydrogens are omitted for clarity.

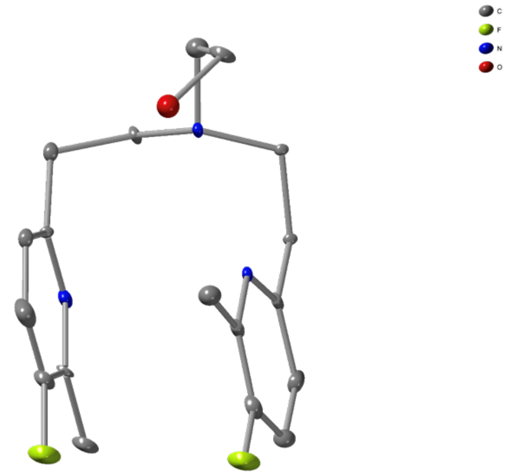

Figure S40. **ORTEP representation of the crystal structure of 6**. Atoms are presented as thermal ellipsoids with 50% probability. Perchlorate counter ions and hydrogens are omitted for clarity.

Table S2: Crystallographic data.

Dataset	Compound 3+ Zn^{2+}	Compound 4 + Zn^{2+}	Compound 5 +Zn^{2+}	Compound 2 + Zn^{2+}	Compound 6
CCDC No.	2084671	2084667	2084668	2084669	2084670
Formula	$C_{14}H_{19}F_2N_3O_3Zn$ + $2ClO_4$ + H_2O	$C_{32}H_{39}F_4N_6O_3$ Zn_2+ $3ClO_4$	$C_{32}H_{36}F_4N_6O_2$ Zn_2+ $2ClO_4$	$C_{28}H_{29}F_4N_6O_2Zn$ + $2ClO_4$	$C_{18}H_{24}F_2N_3O$ + Cl + $1.967H_2O$
Formula weight	597.61	1060.74	942.31	821.84	407.27
Crystal system	Monoclinic	Monoclinic	Monoclinic	Triclinic	Monoclinic
Space group	*P2₁/c*	*P2₁/n*	*P2₁/n*	*P-1*	*P2₁*
Crystal size (mm)	0.143 × 0.064 × 0.056	0.205 × 0.078 × 0.054	0.122 × 0.094 × 0.022	0.120 × 0.095 × 0.090	0.245 × 0.163 × 0.112
Crystal color and shape	Colorless prism	Colorless plate	Colorless plate	Colorless plate	Colorless prism
Temperature (K)	100	100	100	100	100
a (Å)	14.3912(4)	12.34501(3)	12.5355(4)	9.9295(2)	7.7177(1)
b (Å)	10.5859(4)	15.4845(4)	8.4622(3)	10.0220(3)	7.8077(1)
c (Å)	15.6685(5)	21.5589(6)	17.2728(5)	16.9240(4)	65.7902(6)
α (°)	90	90	90	97.113(2)	90
β (°)	111.327(3)	90.515(2)	92.523(3)	94.116(2)	90.004(1)
γ (°)	90	90	90	94.536(2)	90
Volume ($Å^3$)	2223.54(13)	4120.95(12)	1830.49(10)	1660.30(7)	3964.35(8)
Z	4	4	2	2	8
$\rho_{calcd,}$(g cm^{-1})	1.785	1.710	1.710	1.644	1.365
μ (mm^{-1})	1.429	1.452	1,542	0.988	0.234
No. of reflection (unique)	10637 (10637)	79908 (9443)	51836 (5579)	15694 (15694)	88050 (19629)
R_{int}		0.1148	0.0705		0.0410
Completeness to θ (%)	99.5	100.0	100.0	99.0	99.8
Data / restraints / parameters	10637/ 2 / 337	9443 / 4 / 805	5579 / 0 / 353	15694 / 0 / 566	19629 / 2 / 946
Goodness-of-fit on F^2	1.042	1.046	1.037	1.033	1.099
Final R_1 and wR_2 indices [$I > 2\sigma(I)$]	0.0518, 0.1387	0.0543, 0.1179	0.0330, 0.0763	0.0597, 0.1594	0.0578, 0.1237
R_1 and wR_2 indices (all data)	0.0669, 0.1505	0.0696, 0.1240	0.0458, 0.0807	0.0912, 0.1834	0.0766, 0.1336
Largest diff. peak and hole (e/$Å^3$)	1.308, -0.661	1.143, -0.845	0.472, -0.505	1.020, -1.097	0.533, -0.418

L. Supplementary references

1. Zaiss, M.; Bachert, P., Exchange-dependent relaxation in the rotating frame for slow and intermediate exchange – modeling off-resonant spin-lock and chemical exchange saturation transfer. *NMR in Biomedicine* **2013,** *26* (5), 507-518.
2. Zaiss, M.; Zu, Z.; Xu, J.; Schuenke, P.; Gochberg, D. F.; Gore, J. C.; Ladd, M. E.; Bachert, P., A combined analytical solution for chemical exchange saturation transfer and semi-solid magnetization transfer. *NMR in Biomedicine* **2015,** *28* (2), 217-230.
3. Zaiss, M.; Angelovski, G.; Demetriou, E.; McMahon, M. T.; Golay, X.; Scheffler, K., QUESP and QUEST revisited – fast and accurate quantitative CEST experiments. *Magn Reson Med* **2018,** *79* (3), 1708-1721.
